# A phase II randomized clinical trial to assess toxicity and quality of life of breast cancer patients with hypofractionated versus conventional fractionation radiotherapy with regional nodal irradiation in the context of COVID-19 crisis

**DOI:** 10.3389/fonc.2023.1202544

**Published:** 2023-06-14

**Authors:** Gabriel Oliveira Bernardes Gil, Warne Pedro de Andrade, Paulo Henrique Costa Diniz, Farley Soares Cantidio, Izabella Nobre Queiroz, Maria Luísa Braga Vieira Gil, Conceição Aparecida Medeiros Almeida, Paola Palmer Reis Caldeira, Marcos Regalin, Agnaldo Lopes Silva-Filho

**Affiliations:** ^1^ Department of Radiation Oncology, Rede Mater Dei and Hospital da Baleia, Belo Horizonte, Minas Gerais, Brazil; ^2^ Gynecology Department, Universidade Estadual Paulista “Júlio de Mesquita Filho”, Botucatu, São Paulo, Brazil; ^3^ ONCOBIO, Grupo Oncoclinicas, Department of Gynecology and Obstetrics of the School of Medicine of the Federal University of Minas Gerais, Belo Horizonte, Minas Gerais, Brazil; ^4^ Department of Oncology, Rede Mater Dei, Belo Horizonte, Minas Gerais, Brazil; ^5^ Department of Internal Medicine, School of Medicine, Universidade Federal de Minas Gerais, Belo Horizonte, Minas Gerais, Brazil; ^6^ Department of Radiation Oncology, Hospital da Baleia, Belo Horizonte, Minas Gerais, Brazil; ^7^ Department of Radiation Oncology, Rede Mater Dei, Belo Horizonte, Minas Gerais, Brazil; ^8^ Department of Gynecology and Obstetrics of the School of Medicine of the Federal University of Minas Gerais, Belo Horizonte, Minas Gerais, Brazil

**Keywords:** breast cancer, radiation dose hypofractionation, toxicity, breast cancer lymphedema, quality of life

## Abstract

**Purpose:**

This study, conducted during the COVID-19 crisis, primarily aimed to compare the acute toxicity between conventional fractionated radiation therapy (CF-RT) with hypofractionated radiation therapy (HF-RT) among patients who underwent breast-conserving surgery or mastectomy in whom breast or chest wall and regional nodal irradiation (RNI) were indicated. The secondary endpoints were both acute and subacute toxicity, cosmesis, quality of life, and lymphedema features.

**Methods:**

In this open and non-inferiority randomized trial, patients (n = 86) were randomly allocated 2:1 in the CF-RT arm (n = 33; 50 Gy/25 fractions ± sequential boost [10 Gy/5 fractions]) versus the HF-RT arm (n = 53; 40 Gy/15 fractions ± concomitant boost [8 Gy/15 fractions]). Toxic effects and cosmesis evaluation used the Common Terminology Criteria for Adverse Events, version 4.03 (CTCAE) and the Harvard/National Surgical Adjuvant Breast and Bowel Project (NSABP)/Radiation Therapy Oncology Group (RTOG) scale. For the patient-reported quality of life (QoL), the European Organisation for Research and Treatment of Cancer quality of life questionnaire (EORTC QLQ-C30) and the breast cancer-specific supplementary questionnaire (QLQ-BR23) were used. Lymphedema was assessed by comparing volume differences between the affected and contralateral arms using the Casley–Smith formula.

**Results:**

Grade 2 and grade 3 dermatitis were lower with HF-RT than with CF-RT (28% *vs.* 52%, and 0% *vs.* 6%, respectively; p = 0.022). HF-RT had a lower rate of grade 2 hyperpigmentation (23% *vs.* 55%; p = 0.005), compared to CF-RT. No other differences in overall rates of physician-assessed grade 2 or higher and grade 3 or higher acute toxicity between HF-RT and CF-RT were registered. There was no statistical difference between groups regarding cosmesis, lymphedema rate (13% *vs.* 12% HF-RT *vs.* CF-RT; p = 1.000), and functional and symptom scales, during both the irradiation period and after 6 months of the end of treatment. The results revealed that the subset of patients up to 65 years or older did not show a statistical difference between both arm fractionation schedules (p > 0.05) regarding skin rash, fibrosis, and lymphedema.

**Conclusion:**

HF-RT was non-inferior to CF-RT, and moderate hypofractionation showed lower rates of acute toxicity, with no changes in quality-of-life outcomes.

**Clinical trial registration:**

ClinicalTrials.gov, identifier NCT 40155531.

## 1 Introduction

Hypofractionated radiation therapy (HF-RT), in which irradiation may be delivered in dose fractions greater than 2 Gy/day, has emerged as an important tool in breast cancer radiation therapy (RT) (1). Previously, the standard RT dose consisted of 50 Gy in 25 fractions, 2 Gy per daily fraction, corresponding to conventional fractionated radiation therapy (CF-RT) (2, 3). However, after the publication of important phase 3 trials, such as START A and START B, the American Society for Radiation Oncology (ASTRO) endorsed this technique in the treatment of breast cancer (4–8) and extended its indication to patients of all ages, irrespective of chemotherapy receipt (9, 10). Nevertheless, despite the comparable long-term local control, equivalent or modestly improved toxicity outcomes, and additional benefits such as convenience and reduced costs, HF-RT incorporation in practice had been slow and varied worldwide (11).

The arguments against the routine adoption of HF-RT for breast cancer are often based on concerns about the underrepresentation of certain patient subgroups in major trials. Additional limiting use includes uncertainties regarding adverse effects of a higher daily fraction on the heart/lung/brachial plexus and paucity of data on the effects of hypofractionation in the regional nodal irradiation (RNI), post-mastectomy, and breast reconstruction setting (12). In 2019, addressing the representativeness of different patient populations, a phase 3 trial showed the non-inferiority of post-mastectomy RNI hypofractionation over the CF-RT schedule after surgery (12).

On March 11, 2020, the World Health Organization declared the COVID-19 outbreak a global pandemic. Public health officials mobilized communities to minimize transmission by self-isolation and social distancing (13). This scenario catalyzed the hypofractionation implementation broadly (14). In this context, we carried out a randomized phase 2 trial with the primary objective of comparing, in our population, the acute toxicity of HF-RT with CF-RT after breast-conserving surgery or mastectomy with RNI, including the internal mammary nodes (IMNs), when indicated. Then, acute and subacute toxicity, cosmesis, quality of life, and lymphedema features, at different times of the patient journey, were also investigated. The hypothesis established was the non-inferiority of the toxicity of the HF-RT arm compared to the CF-RT.

## 2 Methods

This phase II study was approved by the local research ethics committee under the number 51139715.0.0000.5123 and registered in 2019 on ClinicalTrials.gov (NCT04015531). The study was single-center, conducted on Hospital da Baleia, a Brazilian referral tertiary hospital, which performs radiation therapy among patients from Minas Gerais, the second-most populous state in Brazil. Written informed consent was obtained from each participant. The study was conducted in accordance with the Declaration of Helsinki.

### 2.1 Enrollment

Patients were enrolled from November 2019 through May 2022 at Hospital da Baleia, a referral public oncology tertiary center in Belo Horizonte, Minas Gerais, Brazil. The inclusion criteria were female gender, 18 years or older, breast carcinoma, T1-4 with at least one positive lymph node (American Joint Committee on Cancer (AJCC) 8th) (15), mastectomy or breast-conserving surgery with the investigation of sentinel lymph node or axillary dissection. Adjuvant chemotherapy and hormone therapy were performed as local practice. Neoadjuvant chemotherapy and the use of breast implants were allowed in both study groups. Exclusion criteria were compromised margin, concomitant chemotherapy, internal mammary chain (IMC) or supraclavicular fossa lymph node involvement, previous chest RT, collagen disease, bilateral breast cancer, inflammatory carcinoma, concurrent skin treatment with irradiation, distant metastasis, and synchronic malignancy.

### 2.2 Randomization

Patients were randomly allocated to the control arm, CF-RT (50 Gy/25 fractions ± a sequential boost of 10 Gy/5 fractions, over 25–30 days), or the experimental arm, HF-RT (40 Gy/15 fractions ± a concomitant boost of 8 Gy/15 fractions, over 15 days) following breast surgery. The boost was realized in all cases of breast-conserving surgery. Randomization was planned and performed initially through a computer-generated 2:1 allocation (HF-RT *vs.* CF-RT) to preserve the safety, rights, and well-being of trial participants during the prolonged global public health crisis. Thus, we had more patients with a lower number of physician visits and fewer cross-transmission.

### 2.3 Treatment

Free-breathing computed tomography (CT) scans, with 5-mm slice thickness, in a supine position with arms raised over the head and supported by a ramp for immobilization were obtained for simulation. The organs at risk (OARs) and the target volumes were contoured according to the Radiation Therapy Oncology Group (RTOG) atlas (16–18). The planning target volume (PTV) was delineated with a 7-mm expansion from the clinical target volume (CTV) and 5 mm cropped from the skin, excluding the heart of the treatment volume (19). For women who underwent axillary dissection, the nodal irradiation included the ipsilateral axillary level III and supraclavicular nodes. For patients undergoing sentinel node surgery, nodal RT included the ipsilateral axillary level (I, II, and III) and supraclavicular nodes within the portals. Irradiation of the IMNs was performed based on the physician’s discretion, including from the first to third intercostal space.

Three-dimensional conformal radiotherapy (3D-CRT) was performed using 6- to 10-MV photons. The dose fields were normalized in the same way as the three-field technique photon field. No axilla posterior field was permitted. If any part of the heart was included in the tangential fields, a multileaf collimator was used to shield it from the photon fields. The humeral head, larynx, and trachea were also shielded by the multileaf collimator. Dose constraints followed the RTOG 1005 protocol. At least 95% of each PTV was expected to receive >95% of the prescribed dose. The recommended maximum dose point was not greater than 110%.

### 2.4 Follow-up

All patients were evaluated at baseline, weekly during treatment, just at the end and 1, 2, and 6 months after treatment. The treating physician, a specialist in radiation therapy trained for the study procedures, assessed toxic effects and cosmesis using the Common Terminology Criteria for Adverse Events, version 4.03 (CTCAE v. 4.03). The Harvard/National Surgical Adjuvant Breast and Bowel Project (NSABP)/RTOG scale and pictures were taken at each moment (20–22). Patient-reported quality of life (QoL) was obtained at the first medical appointment, at the end of irradiation, and 1, 2, and 6 months after RT, using the Portuguese-validated versions of the European Organisation for Research and Treatment of Cancer (EORTC) QLQ-30 and the breast cancer-specific QLQ-BR23 applied questionnaires (20, 21, 23). The treating physician did not participate in the data analysis, which was performed by an independent committee.

### 2.5 Lymphedema evaluation

Lymphedema was evaluated by measuring the circumference of the affected and contralateral arm using the Casley–Smith volume formula (22, 24). The volume of each arm was estimated by the formula, corresponding to the distance from the wrist to the arm, which was divided by four segments of truncated cones separated every 10 cm, as exemplified by the calculation between segments C1 and C2 below:


$$V_{2}=\;\frac{h\;\times \;\left(C_{1}{\;}^{2}+\;\left(C_{1}\;\times \;C_{2}\right)\;+\;C_{2}{\;}^{2}\right)}{12\;\times \;\pi }\;,\;h=100\;mm$$

considering h = 100 mm a constant, the volume of each arm was estimated as the sum of the truncated cones (22).

The arm volume was the assessment at the first RT visit, during the discharge, and 6 months after the end of irradiation. After the measurement, the data were tabulated in a spreadsheet, with the formula already inserted for automatic calculations. Volume differences (VDs) between the affected arm and the contralateral were used to define lymphedema. VD >10% was classified as lymphedema (25, 26).

### 2.6 Statistical methods

The primary endpoint of this randomized phase II trial was the assessment of acute toxicity, considered from the baseline to the 3 months after RT, comparing the CF-RT regimen versus HF-RT. Acute toxicity fulfills clinical parameters, such as dermatitis, hyperpigmentation, and edema. Secondary outcomes included subacute toxicity, assessment of QoL, cosmesis, and lymphedema of patients treated with irradiation, presented at 6 months after treatment.

The trial was designed to enroll 80 evaluable patients, which yielded 80% power with a one-sided significance level of 0.05 to test the hypothesis that the probability of any grade ≥ 2 acute toxic effect Hyperfractionated whole breast irradiation (HF-WBI) is no more than 10% worse than the probability of CF-RT, assuming a prevalence of any grade ≥ 2 acute toxic outcome of 78% with CF-RT and 47% with HF-RT and a dropout rate of 15% (27).

Descriptive statistics were used to summarize the data. In the evaluation of the categorical variables, absolute and relative frequencies were determined. For the numerical variables, the absolute frequency, mean, and standard deviation were considered. The variables measured by the EORTC QLQ-C30 and QLQ BR23 were modified by linear transformation with scores from 0 to 100, whose high scores represented a high (better) level of functioning/symptoms or a low (worse) level. A normality test (Shapiro–Wilk) was performed for each continuous variable. The comparative analysis of categorical variables between the control and experimental groups was performed using the chi-square test and Fisher’s exact test, while numerical variables were compared using the Mann–Whitney U test. The intergroup comparison of the EORTC QLQ-C30 was performed using the generalized estimating equation (GEE) method, known as an extension of generalized linear models. All analyses used two-sided α = 0.05 and were performed using the R software version 4.1.2.

## 3 Results

**Figure 1:**
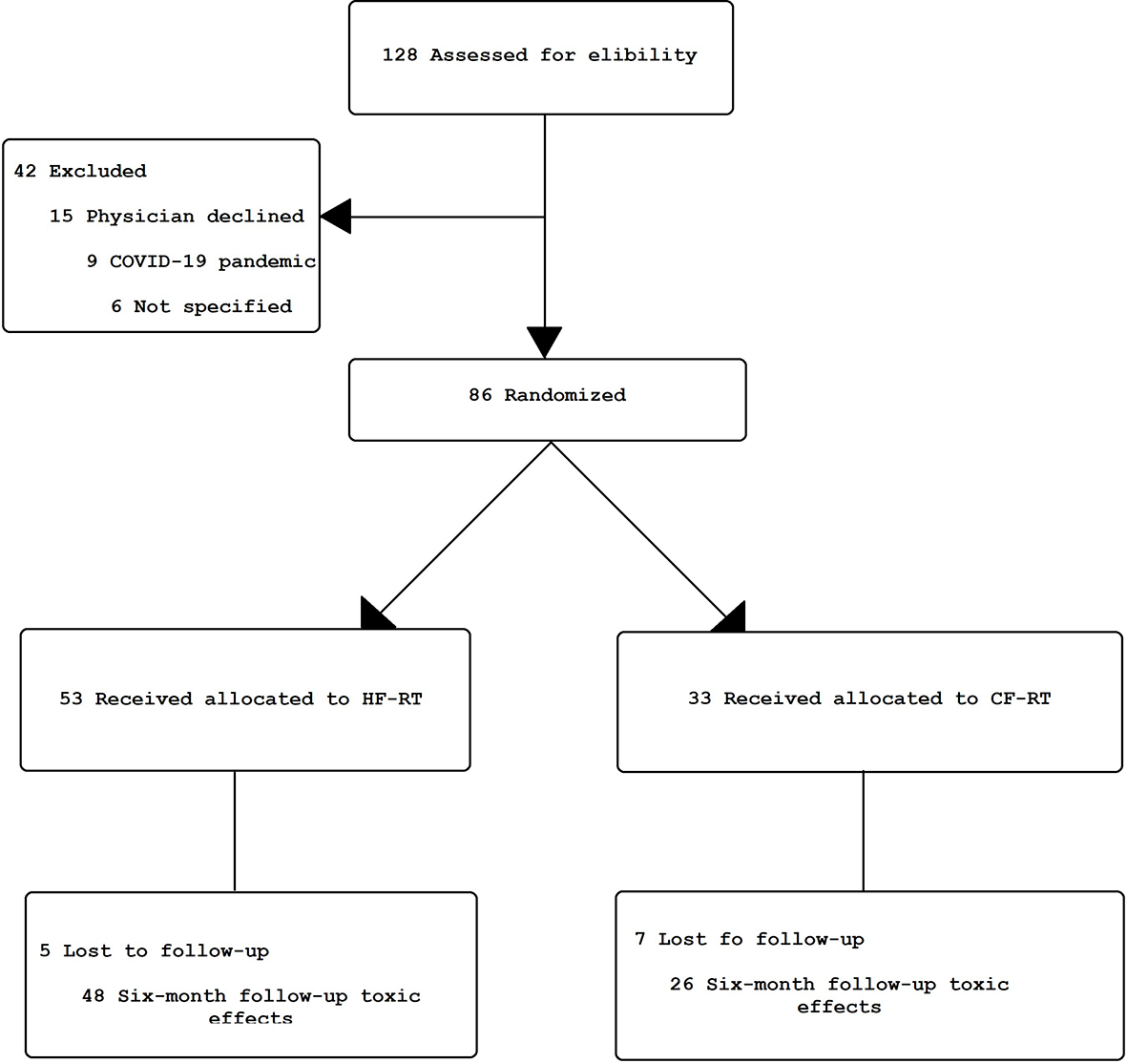
Clinical trial flowchart. HF-RT, hypofractionated radiation therapy; CF-RT, conventional fractionated radiation therapy.

Between November 2019 and February 2022, 128 patients were assessed for eligibility. A total of 86 women were allocated to the CF-RT (n = 53; 62%) or HF-RT arm (n = 33; 38%) (**Figure 1**). The mean age was 57 years (range, 25–91), and patients were self-declared white (30% × 41%), mixed ethnic group (37% × 44%), and black (33% × 15%) in the CF-RT and HF-RT arms. Regarding educational degrees, the majority of patients held a low schooling level (**Supplementary Material**). Most patients underwent breast-conserving surgery (CF-RT 73% *vs.* HF-RT 72%). Mean breast volume, measured using CTV volume, was greater than 1,100 cc (CF-RT 1196 cc *vs.* HF-RT 1,224 cc). Post-mastectomy breast reconstruction accounted for 17% and 12%, respectively, in the HF-RT and CF-RT groups. Of the patients, 56% underwent axillary dissection and 44% sentinel lymph node evaluation, with a mean of 10 lymph nodes removed and two positive lymph nodes. Almost two-thirds of patients had N1 staging, and the mean tumor size in this investigation was 3 cm, with 70% and 30% of staging II and III, respectively (**Supplementary Material**). Most of the women had invasive ductal carcinoma (IDC) and positive hormone receptors. In total, endocrine therapy was used in 67% of the women. Tamoxifen was the main drug, followed by anastrozole. More than two-thirds of patients received either neoadjuvant or adjuvant chemotherapy (CF-RT 72% *vs.* HF-RT 79%). IMC irradiation was performed in 14% (CF-RT 15% *vs.* HF-RT 13%) (**Supplementary Material**).

There were no differences in overall rates of any physician-assessed grade 2 or higher and grade 3 or higher acute toxicity between HF-RT and CF-RT. For specific acute toxicity effects, patients treated with HF-RT *vs.* CF-RT had a lower rate of grade 2 hyperpigmentation (23% *vs.* 55%; p = 0.005). The skin rash grade 2 and grade 3 dermatitis were lower with HF-RT than with CF-RT (28% *vs.* 52%, and 0% *vs.* 6%, respectively; p = 0.022). Most of the irradiated breasts showed no alteration compatible with fibrosis of the skin and subcutaneous tissue. There was no difference in acute grade 1 or higher for fibrosis and hypopigmentation. According to the esthetics assessment, most of the patients had excellent or good grades in both arms (CF-RT 40% *vs.* HF-RT 47%, and 27% CF-RT *vs.* 34% CF-RT, respectively; p = 0.288) (**Table 1**).

**Table 1:** Physician-reported maximum acute toxic effects.

Acute skin toxicity		CF-RT (N = 33)	HF-RT (N = 53)	p
Skin rash (radiotherapy-associated dermatitis)				
	Grade 0	1 (3%)	4 (8%)	**0.022**
	Grade 1	13 (39%)	34 (64%)	
	Grade 2	17 (52%)	15 (28%)	
	Grade 3	2 (6%)	0 (0%)	
Hyperpigmentation				
	Grade 0	0 (0%)	4 (8%)	**0.005**
	Grade 1	15 (45%)	37 (70%)	
	Grade 2	18 (55%)	12 (23%)	
Hypopigmentation				
	Grade 0	21 (64%)	32 (60%)	0.912
	Grade 1	11 (33%)	20 (38%)	
	Grade 2	1 (3%)	1 (2%)	
Induration/fibrosis of skin or subcutaneous tissue				
	Grade 0	21 (64%)	30 (57%)	0.854
	Grade 1	11 (33%)	18 (34%)	
	Grade 2	1 (3%)	4 (8%)	
	Grade 3	0 (0%)	1 (2%)	
Fibrosis/cosmetics				
	Grade 0	25 (76%)	34 (64%)	0.499
	Grade 1	4 (12%)	10 (19%)	
	Grade 2	4 (12%)	6 (11%)	
	Grade 3	0 (0%)	3 (6%)	
Deep connective tissue fibrosis				
	Grade 0	25 (76%)	34 (64%)	0.456
	Grade 1	6 (18%)	11 (21%)	
	Grade 2	2 (6%)	8 (15%)	
Any acute toxicity grade 2 or higher				
	No Yes	10 (30%) 23 (70%)	26 (49%) 27 (51%)	0.136
Any acute toxicity grade 3 or higher				
	No Yes	27 (82%) 6 (18%)	48 (91%) 5 (9%)	0.322
Harvard/NSABP/RTOG breast cosmesis grading scale				
	Poor Fair Good Excellent	5 (15%) 6 (18%) 13 (40%) 9 (27%)	2 (4%) 8 (15%) 25 (47%) 18 (34%)	0.288

As defined by the Harvard/NSABP/RTOG grading scale and CTCAE v. 4.03. Cosmesis and acute toxic effects were recorded on a weekly basis during radiation therapy using a structured template that specified these toxic effects and their definitions. Any subsequent toxic effect occurring within 60 days of treatment completion was also included in this analysis. Fisher’s exact test was used for all values except for any grade 2 or higher toxic effect or any grade 3 or higher toxic effect (χ^2^).

HF-RT, hypofractionated radiation therapy; CF-RT, conventional fractionated radiation therapy; NSABP, National Surgical Adjuvant Breast and Bowel Project; RTOG, Radiation Therapy Oncology Group; CTCAE, Common Terminology Criteria for Adverse Events. Bold values means statistically significant.

The comparative analysis of the physician-reported maximum global toxicity, including skin rash, fibrosis, and lymphedema, according to patients aged 65 years or older (**Table 2**), did not show a statistical difference between both arms (p > 0.05).

**Table 2:** Physician-assessed maximum toxic effects at 6 months.

Subacute skin toxicity		CF-RT (N = 26)	HF-RT (N = 48)	p
Skin rash (radiotherapy-associated dermatitis)				
	Grade 0	26 (100%)	45 (94%)	0.548
	Grade 1	0 (0%)	3 (6%)	
Hyperpigmentation				
	Grade 0	7 (27%)	22 (46%)	0.125
	Grade 1	15 (58%)	24 (50%)	
	Grade 2	4 (15%)	2 (4%)	
Hypopigmentation				
	Grade 0	25 (96%)	46 (96%)	1.000
	Grade 1	1 (4%)	2 (4%)	
Induration/fibrosis of skin or subcutaneous tissue				
	Grade 0	21 (81%)	33 (69%)	0.792
	Grade 1	4 (15%)	11 (23%)	
	Grade 2	1 (4%)	3 (6%)	
	Grade 3	0 (0%)	1 (2%)	
Fibrosis/cosmetics				
	Grade 0	22 (84%)	37 (77%)	0.821
	Grade 1	2 (8%)	6 (13%)	
	Grade 2	2 (8%)	3 (6%)	
	Grade 3	0 (0%)	2 (4%)	
Deep connective tissue fibrosis				
	Grade 0	23 (88%)	39 (81%)	0.793
	Grade 1	2 (8%)	5 (11%)	
	Grade 2	1 (4%)	4 (8%)	
Harvard/NSABP/RTOG breast cosmesis grading scale				
	Poor Fair Good Excellent	0 (0%) 1 (4%) 10 (38%) 15 (58%)	1 (2%) 5 (10%) 11 (23%) 31 (65%)	0.432

HF-RT, hypofractionated radiation therapy; CF-RT, conventional fractionated radiation therapy; NSABP, National Surgical Adjuvant Breast and Bowel Project; RTOG, Radiation Therapy Oncology Group; CTCAE, Common Terminology Criteria for Adverse Events.

As defined by the Harvard/NSABP/RTOG grading scale and CTCAE v. 4.03. Cosmesis and acute toxic effects were recorded 6 months after radiation therapy using a structured template that specified these toxic effects and their definitions. Any subsequent toxic effect occurring 6 months after the treatment completion was also included in this analysis.

The Fisher’s exact test was used for all values except for any grade 2 or higher toxic effect or any grade 3 or higher toxic effect (χ^2^).

**Figure 2:**
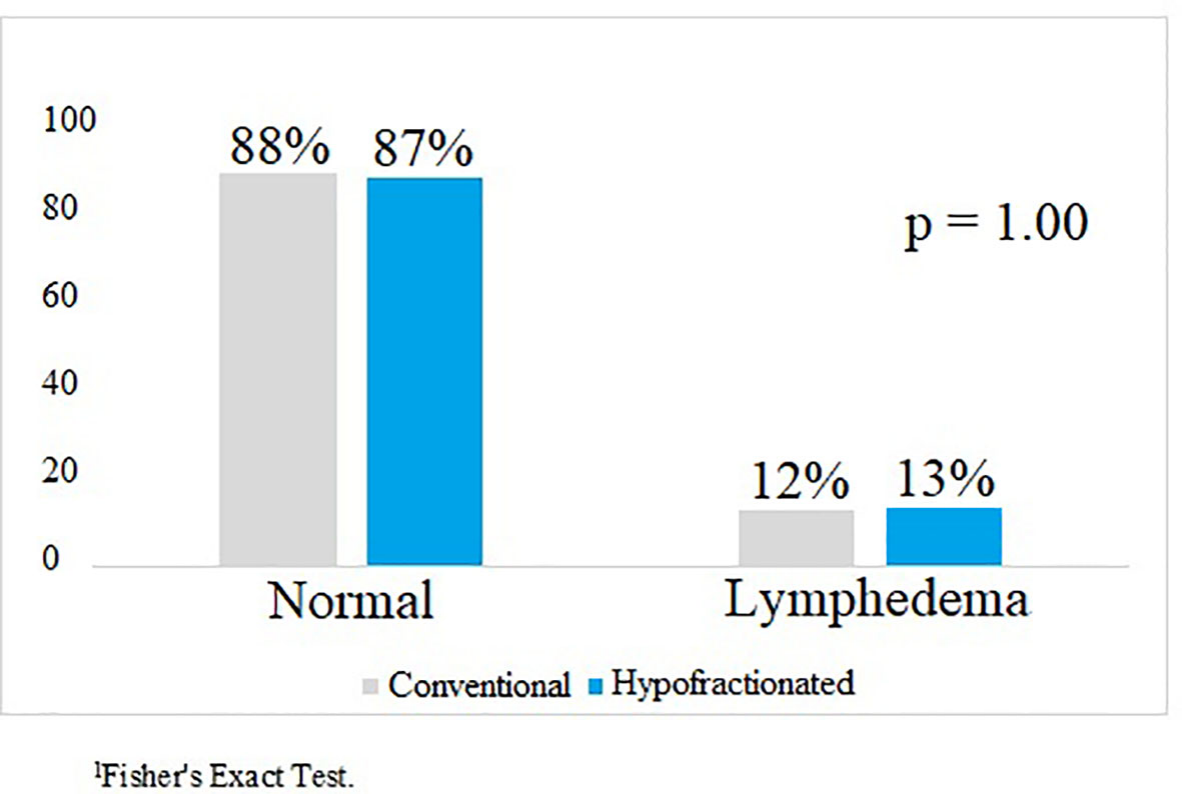
Comparison of lymphedema by randomization arm 6 months after treatment.

A total of 74 patients were evaluated for 6-month toxicity effects. There was no difference between arms regarding subacute toxicity, including the Harvard/NSABP/RTOG cosmesis scale (**Table 3**). There was no statistically significant difference in the rate of lymphedema after 6 months of treatment between the two RT fractionation groups (13% *vs.* 12% HF-RT *vs.* CF-RT, respectively; p = 1.000) (**Figure 2**). There were no reports of acute or subacute grade 4 toxicity, no symptomatic pulmonary toxicity, ischemic cardiac event, capsular contracture, rib fracture, brachial plexopathy, deaths, or distant metastases during the analyzed period. There was no statistical difference between the CF-RT and HF-RT arms from baseline to 6 months after treatment in functional and symptom scales of the QLQ-C30 questionnaire (**Table 4**). As detailed in **Table 5**, analysis of the QLQ-BR23 questionnaire showed no difference in symptom and functional scales between CF-RT and HF-RT groups.

**Table 3:** Mean baseline and 1-, 2-, and 6-month EORTC QLQ-C30 scale by randomization arm.

	CF-RT	HF-RT	
	Mean (SD)	Mean (SD)	p-Value
Baseline			
Fatigue	16 (26)	13 (19)	0.549
Nausea and vomiting	10 (22)	4 (12)	0.206
Pain	24 (31)	14 (23)	0.111
Dyspnea	12 (26)	8 (23)	0.467
Insomnia	28 (40)	16 (30)	0.117
Loss of appetite	13 (31)	11 (27)	0.706
Constipation	23 (35)	6 (20)	0.07
Diarrhea	3 (13)	3 (11)	0.847
Financial difficulties	23 (35)	16 (30)	0.298
One-month follow-up			
Fatigue	20 (29)	13 (20)	0.175
Nausea and vomiting	10 (24)	7 (13)	0.421
Pain	20 (30)	19 (23)	0.746
Dyspnea	18 (31)	9 (19)	0.096
Insomnia	26 (41)	19 (36)	0.394
Loss of appetite	15 (33)	10 (23)	0.392
Constipation	16 (30)	9 (26)	0.217
Diarrhea	6 (21)	3 (9)	0.367
Financial difficulties	23 (39)	21 (35)	0.778
Two-month follow-up			
Fatigue	15 (25)	15 (22)	0.976
Nausea and vomiting	3 (9)	5 (14)	0.329
Pain	23 (32)	19 (25)	0.544
Dyspnea	14 (31)	13 (29)	0.924
Insomnia	26 (40)	19 (36)	0.481
Loss of appetite	11 (28)	13 (28)	0.717
Constipation	14 (32)	10 (23)	0.558
Diarrhea	4 (19)	3 (14)	0.813
Financial difficulties	22 (38)	18 (34)	0.68
Six-month follow-up			
Fatigue	13 (21)	18 (27)	
Nausea and vomiting	4 (10)	10 (23)	
Pain	21 (31)	23 (30)	
Dyspnea	9 (24)	13 (26)	
Insomnia	26 (36)	30 (40)	
Loss of appetite	18 (32)	12 (25)	
Constipation	15 (34)	16 (32)	
Diarrhea	4 (20)	6 (20)	
Financial difficulties	14 (29)	16 (31)	

HF-RT, hypofractionated radiation therapy; CF-RT, conventional fractionated radiation therapy; SD, standard deviation; EORTC QLQ-C30, European Organisation for Research and Treatment of Cancer quality of life questionnaire—core questionnaire/Portuguese (Brazil).

p-Value from Mann–Whitney test.

**Table 4:** Mean baseline and 1-, 2-, and 6-month EORTC QLQ-BR23 scale by randomization arm.

Index	CF-RT	HF-RT	p-Value
	Mean (SD)	Mean (SD)	
Baseline			
Functional scales			
Body image	76 (26)	77 (27)	0.808
Sexual functioning	74 (31)	79 (29)	0.45
Sexual pleasure	70 (35)	75 (29)	0.417
Future perspective	73 (36)	77 (29)	0.583
Symptom scales			
Side effects of systemic therapy	26 (22)	24 (16)	0.757
Breast symptoms	30 (29)	19 (26)	0.066
Arm symptoms	24 (26)	17 (21)	0.179
Upset by hair loss	14 (33)	17 (34)	0.699
One-month follow-up			
Functional scale			
Body image	76 (32)	75 (26)	0.941
Sexual functioning	78 (31)	73 (32)	0.526
Sexual pleasure	62 (39)	75 (29)	0.087
Future perspective	76 (30)	76 (29)	0.855
Symptom scales			
Side effects of systemic therapy	24 (21)	20 (15)	0.343
Breast symptoms	20 (27)	16 (20)	0.399
Arm symptoms	20 (22)	15 (19)	0.307
Upset by hair loss	14 (33)	12 (29)	0.774
Two-month follow-up			
Functional scales			
Body image	73 (35)	76 (28)	0.811
Sexual functioning	78 (36)	80 (32)	0.878
Sexual pleasure	71 (36)	68 (36)	0.762
Future perspective	78 (35)	79 (32)	0.947
Symptom scales			
Side effects of systemic therapy	23 (21)	22 (19)	0.966
Breast symptoms	15 (21)	12 (20)	0.558
Arm symptoms	18 (24)	18 (24)	0.995
Upset by hair loss	16 (33)	15 (35)	0.855
Six-month follow-up			
Body image	79 (29)	82 (22)	0.676
Sexual functioning	78 (30)	76 (33)	0.608
Sexual pleasure	69 (39)	70 (35)	0.818
Future perspective	78 (31)	76 (35)	0.578
Symptom scales			
Side effects of systemic therapy	23 (25)	19 (20)	0.864
Breast symptoms	16 (25)	16 (25)	0.612
Arm symptoms	19 (25)	18 (22)	0.939
Upset by hair loss	12 (33)	3 (10)	0.353

HF-RT, hypofractionated radiation therapy; CF-RT, conventional fractionated radiation therapy; SD, standard deviation; EORTC QLQ-BR23, European Organisation for Research and Treatment of Cancer quality of life questionnaire—Breast Module/Portuguese (Brazil).

p-Value from Mann–Whitney test.

**Table 5:** Physician-reported maximum global toxicity according to patients up to 65 years or older.

Global toxicity		<65 years old (N = 60)	≥65 years old (N = 26)	p
Skin rash (radiotherapy-associated dermatitis)				
	Grade 0	5 (8%)	0 (0%)	0.133
	Grade 1	31 (52%)	16 (62%)	
	Grade 2	22 (37%)	10 (38%)	
	Grade 3	2 (3%)	0 (0%)	
Hyperpigmentation				
	Grade 0	3 (5%)	1 (4%)	0.615
	Grade 1	34 (57%)	18 (69%)	
	Grade 2	23 (38%)	7 (27%)	
Hypopigmentation				
	Grade 0	33 (55%)	20 (77%)	0.054
	Grade 1	26 (43%)	5 (19%)	
	Grade 2	1 (2%)	1 (4%)	
Induration/fibrosis of skin or subcutaneous tissue				
	Grade 0	36 (60%)	15 (58%)	0.950
	Grade 1	20 (33%)	9 (34%)	
	Grade 2	3 (5%)	2 (8%)	
	Grade 3	1 (2%)	0 (0%)	
Fibrosis/cosmetics				
	Grade 0	42 (70%)	17 (66%)	0.649
	Grade 1	9 (15%)	5 (19%)	
	Grade 2	6 (10%)	4 (15%)	
	Grade 3	3 (5%)	0 (0%)	
Deep connective tissue fibrosis				
	Grade 0	43 (72%)	16 (62%)	0.581
	Grade 1	11 (18%)	6 (23%)	
	Grade 2	6 (10%)	4 (15%)	
Lymphedema				
	Lymphedema	7 (12%)	3 (12%)	1.000
	Normal	53 (88%)	23 (88%)	

Fisher’s exact test.

## 4 Discussion

While breast HF-RT has been extensively studied, the use of HF in the setting of RNI and post-mastectomy remains more controversial (28). In this prospective, randomized trial, we evaluated the acute and subacute toxicity of HF-RT versus CF-RT after breast-conserving surgery or mastectomy with RNI. A particularly important finding of this study is the acute more favorable toxic outcome with the use of HF-RT in the RNI scenario. Specifically, the incidence of acute grade 2 hyperpigmentation was 32% lower in patients treated with HF-RT than with CF-RT. In addition, acute skin rash grade 2 and grade 3 dermatitis were significantly lower in the HF-RT arm. Both groups showed similar rates of other acute complications such as hypopigmentation and fibrosis of skin or subcutaneous tissue. We observed that HF-RT was similar to CF-RT concerning adverse physician-reported toxic effects 6 months after RT.

Based on long-term results from randomized trials, the evidence supports HF-RT for patients with early-stage, node-negative breast cancer aged >50 years after breast-conserving surgery (BCS). These patients should routinely receive HF-RT regimens of 40–42.6 Gy in 15–16 fractions (8–10, 29). The UK trials have been demonstrating that other even more abbreviated hypofractionated regimens for whole-breast radiation therapy (WBRT) can be delivered. The FAST trial found the dose of 28.5 Gy to be comparable to the 50-Gy arm and significantly milder in toxicity than the 30-Gy arm (30). Sequentially, in the FAST FORWARD trial, 26 Gy in five fractions over 1 week was non-inferior to the standard of 40 Gy in 15 fractions for local tumor control and is as safe in terms of normal tissue effects up to 5 years (31).

Consistent with our findings, 864 women who received locoregional radiotherapy in START trials showed no significant difference in acute toxicity between HF-RT and CF-RT groups (9, 32). Also, in the Chinese large-scale randomized trial directly comparing post-mastectomy with RNI, the HF-RT had less frequent grade 3 acute skin toxicity than the CF-RT arm, 3% *vs.* 8% p< 0.0001 (12). Furthermore, in the MD Anderson trial, maximum physician-reported acute dermatitis was lower in the HF-RT arm (36% *vs.* 69%; p< 0.001).

To our knowledge, this is the first Latin American randomized trial to report acute and subacute breast radiation toxicity between hypofractionation and conventional fractionation. Unlike the majority of the published trials, our population consisted predominantly of self-declared black or mixed ethnicity and had low educational levels (5, 29, 33).

Like other studies performed, we face great challenges due to the COVID-19 pandemic. Most of the time, we deal with the toughest moments of the pandemic. As it was impossible to postpone the treatment or to convert the physical appointment into video visits, we decide to adjust the allocation proportion to allocate a higher number of patients in the HF-RT, as reported above (34, 35). This shift followed the recommendations at that time, with emerging data suggesting no differences in efficacy or toxicity with HF-RT and CF-RT (36, 37).

Regarding the radiation fields, in the Royal Marsden Hospital (RMH) trial, START A, and START B, approximately 21%, 14%, and 7% of the patients received RNI, respectively (4, 38–41). Even though in the Chinese study all patients received level III and supraclavicular fossa nodal irradiation, there was no target volume for axilla and IMC (12). In our study, women with no axillary dissection received RT to levels I, II, and III and supraclavicular fossa, while in those who underwent the lymphadenectomy, the target volume included only the supraclavicular region and level III. IMC irradiation was performed in 15% of patients in the CF-RT arm versus 13% in the HF-RT arm. The randomized trials did not include the internal mammary chain in the target volumes. Despite some studies suggesting equivalent levels of acute and late toxicity, it is not possible to exclude the possibility of increased pulmonary, costal arch, and heart toxicity with hypofractionated radiotherapy (42). No pulmonary toxicity has been observed in patients with IMC irradiation, although we consider that a larger trial with long-term follow-up is required.

Breast reconstruction is performed to restore the breast shape after mastectomy and improves QoL (43). However, post-mastectomy radiation therapy (PMRT) can lead to increased complications of the reconstructed breast (44). There is a paucity of data about how HF-RT affects breast-related complications after breast reconstruction. Kim and colleagues conducted a retrospective investigation of the impact of PMRT with conventional *vs.* hypofractionated settings and detected no difference in the occurrence of any or major breast-related complications between the two fractionations (45). In our trial, we had a small number of patients who underwent breast reconstruction, 12% and 17% in the CF-RT and HF-RT, respectively, and no difference was demonstrated between them. There was no implant failure reported. We look forward to a longer follow-up that could elucidate potential related complications. Current trials are evaluating HF-RT with reconstruction (Alliance221505/NCT03414970; FABREC Trial/NCT03422003).

The tumor bed boost dose was investigated in the EORTC boost trial. The results showed local control improvement, although there was an increased risk of fibrosis (46, 47). The use of a simultaneous integrated boost (SIB) during the whole-breast treatment has several theoretical dosimetric advantages and a more convenient treatment schedule. The dose can be reduced for the remaining breast as well as for OARs. The hypofractionated boost (HF-boost) has not been extensively investigated; however, emerging data suggested that it may be effective and safe. One Chinese study with 185 patients evaluated CF-RT with 50 Gy in 25 fractions followed by a sequential boost of 10 Gy in 5 fractions versus HF-RT with 42.56 Gy in 16 fractions with a SIB up to 48 Gy in 16 fractions. After 2 years, no difference in skin toxicity or cosmetic outcomes between the two arms was detected. Furthermore, the authors highlighted the possibility of hypofractionation with a concomitant boost as a valuable choice to recommend suitable candidates during the COVID-19 epidemic, as we did in our study (48). These findings were consistent with our study, in which all patients undergoing BCS received a boost (concurrent in the HF-RT arm versus sequential in the CF-RT arm), and there was no difference in acute toxicity, fibrosis, or worsening of cosmesis over the 6-month follow-up.

Axillary lymph node dissection and adjuvant radiotherapy are risk factors for lymphedema related to breast cancer (49, 50). The literature has investigated a wide variety of methods for evaluating limb volume when lymphedema is diagnosed. Options include bioelectrical impedance analysis (BIA), tape measurement, perometry, and water displacement. In our trial, lymphedema was evaluated by arm-treated volume measurement in comparison to the contralateral arm (51, 52). In the Indian randomized investigation with CF-RT versus HF-RT at a median follow-up of 20 months, lymphedema was not observed at 88% in conventional irradiation and 86% in hypofractionation (53). A cohort of 1,640 breast cancer patients receiving post-mastectomy radiotherapy found lymphedema in four patients in CF-RT (1%) and four patients in HF-RT (1%), with no statistically significant difference between the schedules (54). Our lymphedema evaluation was performed from the baseline to 6 months after the treatment, and there was no statistically significant difference between arms. As lymphedema is considered a late toxicity effect of radiation therapy, a longer time of follow-up for our patients may be necessary.

Health-related quality of life is considered an important endpoint in cancer clinical trials (55, 56). There are scarce data available to describe patient-reported outcomes of hypofractionation in comparison to conventional fractionation. Jagsi and colleagues present a study with academic and community radiation oncology centers showing higher rates of fatigue 30% *vs.* 19%, p = 0.02, and self-reported moderate/severe pain, 41% *vs.* 24%, p = 0.003, respectively, to the CF-RT versus HF-RT (57). The MD Anderson trial reported less fatigue in patients randomized to the HF-RT group (0% *vs.* 6%; p = 0.01) and less lack of energy (23% *vs.* 39%; p< 0.001) *vs.* the CF-RT group (27). The results of the abovementioned studies conflict with our trial. The QLC-C30 and QLC-BR23 scales were used to assess many factors. No difference was detected in all quality-of-life domains between arms. Nevertheless, it is important to mention that differences in the toxicity profile compared to our trial may be due to a limited number of patients enrolled in the present study, which might be unable and underpowered to detect smaller differences.

Our results revealed that even the subset of patients up to 65 years or older did not show a statistical difference between both arm fractionation schedules (p > 0.05) regarding skin rash, fibrosis, and lymphedema. Hypofractionation is more beneficial for frail and older patients because it reduces the need for transportation and increases their adherence, as verified by other studies (58, 59). Nevertheless, since there are a small number of elderly patients over 70 years old in our research, more studies should be conducted to investigate this finding.

This trial has some limitations. First, our study has a small sample size and a short-term follow-up period for late toxicity. Second, our study was carried out at a single center. Third, it was not double-blind. Fourth, overall survival data and local recurrence outcomes are absent. However, in this study, patients were selected by intention-to-treat analysis, and this analysis may stimulate more future research for these purposes. Additionally, our findings add to the evidence for HF-RT, which would help in therapeutic decisions even after the pandemic period.

## 5 Conclusions

In this randomized phase 2 study, HF-RT showed a lower frequency of skin rash and global acute and subacute toxicity when compared to CF-RT. There was a higher incidence of skin rash and hyperchromia in the control group. Due to the limitations of this analysis, more randomized phase 3 studies with a larger number of patients and a longer follow-up period are needed to better evaluate and compare toxicity.

## Manuscript formatting

### Headings

◼ Hypofractionated radiation therapy has irradiation dose fractions greater than 2 Gy/day.
◼ Conventional fractionation has daily radiation doses of 1.8–2 Gy.
◼ The primary endpoint of this randomized phase II trial was the assessment of acute toxicity.
◼ Secondary endpoints were subacute toxicity, assessment of QoL, cosmesis, and lymphedema.
◼ Skin rash grade 2 and grade 3 dermatitis were lower with HF-RT than with CF-RT.
◼ HF-RT was non-inferior with a lower frequency of skin rash and global acute and subacute toxicity when compared to CF-RT.

## Equations


$$V_{2}=\;\frac{h\;\times \;\left(C_{1}{\;}^{2}+\;\left(C_{1}\;\times \;C_{2}\right)\;+\;C_{2}{\;}^{2}\right)}{12\;\times \;\pi }\;,\;h=100\;mm$$

## Data availability statement

The datasets presented in this study can be found in online repositories. The names of the repository/repositories and accession number(s) can be found in the article/**Supplementary material**.

## Ethics statement

The studies involving human participants were reviewed and approved by 5123 – Hospital da Baleia/Fundação Benjamin Guimarães. The patients/participants provided their written informed consent to participate in this study. Written informed consent was obtained from the individual(s) for the publication of any potentially identifiable images or data included in this article.

## Author contributions

GOBG, PHCD, ALSF contributed to the study concept and design. All the authors performed the acquisition, analysis, and interpretation of data. All authors contributed to the article and approved the submitted version.

## References

[1] Yarnold J. Changes in radiotherapy fractionation-breast cancer. Br J Radiol (2019) 92(1093):1–8. doi: 10.1259/bjr.20170849 PMC6330077 29345152

[2] Fisher B Anderson S Bryant J Margolese RG Deutsch M Fisher ER. Twenty-year follow-up of a randomized trial comparing total mastectomy, lumpectomy, and lumpectomy plus irradiation for the treatment of invasive breast cancer. New Engl J Med (2002) 347(16):1233–41. doi: 10.1056/NEJMoa022152 12393820

[3] Margenthaler JA Dietz JR Chatterjee A. The landmark series: breast conservation trials (including oncoplastic breast surgery). Ann Surg Oncol (2021) 28(4):2120–7. doi: 10.1245/s10434-020-09534-y 33521897

[4] Yarnold J Ashton A Bliss J Homewood J Harper C Hanson J. Fractionation sensitivity and dose response of late adverse effects in the breast after radiotherapy for early breast cancer: long-term results of a randomised trial. Radiother Oncol (2005) 75(1):9–17. doi: 10.1016/j.radonc.2005.01.005 15878095

[5] Group TST. The UK standardisation of breast radiotherapy (START) trial a of radiotherapy hypofractionation for treatment of early breast cancer: a randomised trial, Lancet Oncol (2008) 9(4):331. doi: 10.1016/S1470-2045(08)70077-9 18356109 PMC2323709

[6] Whelan TJ Pignol JP Levine MN Julian JA MacKenzie R Parpia S. Long-term results of hypofractionated radiation therapy for breast cancer. New Engl J Med (2010) 362(6):513–20. doi: 10.1056/NEJMoa0906260 20147717

[7] Smith BD Bentzen SM Correa CR Hahn CA Hardenbergh PH Ibbott GS. Fractionation for whole breast irradiation: an American society for radiation oncology (ASTRO) evidence-based guideline. Int J Radiat Oncol Biol Phys (2011) 81(1):59–68. doi: 10.1016/j.ijrobp.2010.04.042 20638191

[8] Agrawal RK Aird EGA Barrett JM Barrett-Lee PJ Bentzen SM Bliss JM. The UK standardisation of breast radiotherapy (START) trial b of radiotherapy hypofractionation for treatment of early breast cancer: a randomised trial. Lancet (2008) 371(9618):1098. doi: 10.1016/S0140-6736(08)60348-7 18355913 PMC2277488

[9] Haviland JS Owen JR Dewar JA Agrawal RK Barrett J Barrett-Lee PJ. The UK standardisation of breast radiotherapy (START) trials of radiotherapy hypofractionation for treatment of early breast cancer: 10-year follow-up results of two randomised controlled trials. Lancet Oncol (2013) 14(11):1086–94. doi: 10.1016/S1470-2045(13)70386-3 24055415

[10] Smith BD Bellon JR Blitzblau R Freedman G Haffty B Hahn C. Radiation therapy for the whole breast: executive summary of an American society for radiation oncology (ASTRO) evidence-based guideline. Pract Radiat Oncol (2018) 8(3):145–52. doi: 10.1016/j.prro.2018.01.012 29545124

[11] Wang EH Mougalian SS Soulos PR Rutter CE Evans SB Haffty BG. Adoption of hypofractionated whole-breast irradiation for early-stage breast cancer: a national cancer data base analysis. Int J Radiat Oncol Biol Phys (2014) 90(5):993–1000. doi: 10.1016/j.ijrobp.2014.06.038 25149661

[12] Wang SL Fang H Song YW Wang WH Hu C Liu YP. Hypofractionated versus conventional fractionated postmastectomy radiotherapy for patients with high-risk breast cancer: a randomised, non-inferiority, open-label, phase 3 trial. Lancet Oncol (2019) 20(3):352–60. doi: 10.1016/S1470-2045(18)30813-1 30711522

[13] Ferguson NM Laydon D Nedjati-Gilani G Imai N Ainslie K Baguelin M. Impact of non-pharmaceutical interventions (NPIs) to reduce COVID-19 mortality and healthcare demand. Imperial College COVID-19 Response Team doi: 10.25561/77482

[14] Al-Rashdan A Roumeliotis M Quirk S Grendarova P Phan T Cao J. Adapting radiation therapy treatments for patients with breast cancer during the COVID-19 pandemic: hypo-fractionation and accelerated partial breast irradiation to address world health organization recommendations. Adv Radiat Oncol (2020) 5(4):575–6. doi: 10.1016/j.adro.2020.03.011 PMC7194663 32363244

[15] Giuliano AE Edge SB Hortobagyi GN. Eighth edition of the AJCC cancer staging manual: breast cancer. Ann Surg Oncol (2018) 25(7):1783–5. doi: 10.1245/s10434-018-6486-6 29671136

[16] Breast Contouring RADCOMP Consortium. RADCOMP breast atlas committee. (2016). Available at: https://www.nrgoncology.org/About-Us/Center-for-Innovation-in-Radiation-Oncology/Breast-Cancer/RADCOMP-Breast-Atlas

[17] Duane F Aznar MC Bartlett F Cutter DJ Darby SC Jagsi R. A cardiac contouring atlas for radiotherapy. Radiother Oncol (2017) 122(3):416–22. doi: 10.1016/j.radonc.2017.01.008 PMC5356506 28233564

[18] RADCOMP breast atlas v.3 - bigreduced (2023). Available at: https://www.nrgoncology.org/About-Us/Center-for-Innovation-in-Radiation-Oncology/Breast-Cancer/RADCOMP-Breast-Atlas

[19] Vicini FA Winter K Freedman GM Arthur DW Hayman JA Rosenstein BS. RTOG 1005: A phase iii trial of hypo fractionated whole breast irradiation with concurrent boost vs. conventional whole breast irradiation plus sequential boost following lumpectomy for high risk early-stage breast cancer. Int J Radiat Oncol Biol Phys (2022) 114(3):S1. doi: 10.1016/j.ijrobp.2022.07.2320

[20] Sprangers MA Groenvold M Arraras JI Franklin J te Velde A Muller M. The European organization for research and treatment of cancer breast cancer-specific quality-of-life questionnaire module: first results from a three-country field study. J Clin Oncol (1996) 14(10):2756–68. doi: 10.1200/JCO.1996.14.10.2756 8874337

[21] M K MG D M. Quality of life of Danish women: population-based norms of the EORTC QLQ-C30. Qual Life Res (1997) 6(1):27–34. doi: 10.1023/A:1026461310761 9062439

[22] Casley-Smith JR. Measuring and representing peripheral oedema and its alterations. Lymphology (1994) 27(2):56–70. 8078362

[23] Alessandra F Michels S Maria I Dias De Oliveira R Ii L Do M. Validity, reliability and understanding of the EORTC-C30 and EORTC-BR23, quality of life questionnaires specific for breast cancer. Rev Bras Epidemiol (2013) 16(2):352–63. doi: 10.1590/S1415-790X2013000200011 24142007

[24] Tewari N Gill PG Bochner MA Kollias J. Comparison of volume displacement versus circumferential arm measurements for lymphoedema: implications for the SNAC trial. ANZ J Surg (2008) 78(10):889–93. doi: 10.1111/j.1445-2197.2008.04686.x 18959643

[25] Petrek JA Pressman PI Smith RA. Lymphedema: current issues in research and management. CA Cancer J Clin (2000) 50(5):292–307. doi: 10.3322/canjclin.50.5.292 11075239

[26] National Cancer Institute. Lymphedema (PDQ): health professional version . Available at: https://www.cancer.gov/about-cancer/treatment/side-effects/lymphedema/lymphedema-hp-pdq.2022.

[27] Shaitelman SF Schlembach PJ Arzu I Ballo M Bloom ES Buchholz D. Acute and short-term toxic effects of conventionally fractionated vs hypofractionated whole-breast irradiation. JAMA Oncol (2015) 1(7):931. doi: 10.1001/jamaoncol.2015.2666 26247543 PMC4635441

[28] Vinh-Hung V Nguyen NP Verschraegen C. Hypofractionated nodal irradiation for breast cancer: a case for caution. JAMA Oncol (2019) 5(1):13–4. doi: 10.1001/jamaoncol.2018.5061 30383169

[29] Whelan T MacKenzie R Julian J Levine M Shelley W Grimard L. Randomized trial of breast irradiation schedules after lumpectomy for women with lymph node-negative breast cancer. J Natl Cancer Institute (2002) 94(15):1143–50. doi: 10.1093/jnci/94.15.1143 12165639

[30] Brunt AM Haviland JS Sydenham M Agrawal RK Algurafi H Alhasso A. Ten-year results of fast: a randomized controlled trial of 5-fraction whole-breast radiotherapy for early breast cancer. J Clin Oncol (2020) 38(28):3261–72. doi: 10.1200/JCO.19.02750 PMC7526720 32663119

[31] Murray Brunt A Haviland JS Wheatley DA Sydenham MA Alhasso A Bloomfield DJ. Hypofractionated breast radiotherapy for 1 week versus 3 weeks (FAST-forward): 5-year efficacy and late normal tissue effects results from a multicentre, non-inferiority, randomised, phase 3 trial. Lancet (2020) 395(10237):1613–26. doi: 10.1016/S0140-6736(20)30932-6 PMC7262592 32580883

[32] Agrawal RK Aird EGA Barrett JM Barrett-Lee PJ Bentzen SM Bliss JM. The UK standardisation of breast radiotherapy (START) trial b of radiotherapy hypofractionation for treatment of early breast cancer: a randomised trial Lancet (2008) 371(9618):1098–107. doi: 10.1016/S0140-6736(08)60348-7 PMC2277488 18355913

[33] Shaitelman SF Lei X Thompson A Schlembach P Bloom ES Arzu IY. Three-year outcomes with hypofractionated versus conventionally fractionated whole-breast irradiation: results of a randomized, noninferiority clinical trial. J Clin Oncol (2018) 36(35):3495–503. doi: 10.1200/JCO.18.00317 PMC6286164 30379626

[34] Park JJH Mogg R Smith GE Nakimuli-Mpungu E Jehan F Rayner CR. How COVID-19 has fundamentally changed clinical research in global health. Lancet Glob Health (2021) 9(5):e711–20. doi: 10.1016/S2214-109X(20)30542-8 PMC8049590 33865476

[35] Orkin AM Gill PJ Ghersi D Campbell L Sugarman J Emsley R. Guidelines for reporting trial protocols and completed trials modified due to the COVID-19 pandemic and other extenuating circumstances: the CONSERVE 2021 statement. JAMA (2021) 326(3):257–65. doi: 10.1001/jama.2021.9941 34152382

[36] Dietz JR Moran MS Isakoff SJ Kurtzman SH Willey SC Burstein HJ. Recommendations for prioritization, treatment, and triage of breast cancer patients during the COVID-19 pandemic. the COVID-19 pandemic breast cancer consortium. Breast Cancer Res Treat (2020) 181(3):487. doi: 10.1007/s10549-020-05644-z 32333293 PMC7181102

[37] Meattini I Becherini C Boersma L Kaidar-Person O Marta GN Montero A. European Society for radiotherapy and oncology advisory committee in radiation oncology practice consensus recommendations on patient selection and dose and fractionation for external beam radiotherapy in early breast cancer. Lancet Oncol (2022) 23(1):e21–31. doi: 10.1016/S1470-2045(21)00539-8 34973228

[38] Whelan TJ Pignol J Julian J Grimard L Bowen J Perera F. Long-term results of a randomized trial of accelerated hypofractionated whole breast irradiation following breast conserving surgery in women with node-negative breast cancer. Int J Radiat OncologyBiologyPhysics. (2008) 72(1):S28. doi: 10.1016/j.ijrobp.2008.06.829

[39] Haffty BG. Long-term results of hypofractionated radiation therapy for breast cancer. Yearbook Oncol (2010) 2010:32–3. doi: 10.1016/S1040-1741(10)79575-0

[40] Owen JR Ashton A Bliss JM Homewood J Harper C Hanson J. Effect of radiotherapy fraction size on tumour control in patients with early-stage breast cancer after local tumour excision: long-term results of a randomised trial. Lancet Oncol (2006) 7(6):467–71. doi: 10.1016/S1470-2045(06)70699-4 16750496

[41] START Trialists’ Group Bentzen SM Agrawal RK Aird EGA Barrett JM Barrett-Lee PJ. The UK standardisation of breast radiotherapy (START) trial a of radiotherapy hypofractionation for treatment of early breast cancer: A randomised trial Lancet Oncol (2008) 9(4):331–41. doi: 10.1016/S1470-2045(08)70077-9 PMC2323709 18356109

[42] Freitas NMA Rosa AA Marta GN Hanna SA Hanriot R de M Borges ABB. Recommendations for hypofractionated whole-breast irradiation. Rev Assoc Med Bras (2018) 64(9):770–7. doi: 10.1590/1806-9282.64.09.770 30672995

[43] Jagsi R Li Y Morrow M Janz N Alderman A Graff J. Patient-reported quality of life and satisfaction with cosmetic outcomes after breast conservation and mastectomy with and without reconstruction: results of a survey of breast cancer survivors. Ann Surg (2015) 261(6):1198–206. doi: 10.1097/SLA.0000000000000908 PMC4512928 25654742

[44] Jagsi R Momoh AO Qi J Hamill JB Billig J Kim HM. Impact of radiotherapy on complications and patient-reported outcomes after breast reconstruction. J Natl Cancer Inst (2018) 110(2):157–65. doi: 10.1093/jnci/djx148 PMC6059091 28954300

[45] Kim DY Park E Heo CY Jin US Kim EK Han W. Hypofractionated versus conventional fractionated radiotherapy for breast cancer in patients with reconstructed breast: toxicity analysis. Breast (2021) 55:37–44. doi: 10.1016/j.breast.2020.11.020 33316582 PMC7744765

[46] Bartelink H Horiot JC Poortmans P Struikmans H van den Bogaert W Barillot I. Recurrence rates after treatment of breast cancer with standard radiotherapy with or without additional radiation. New Engl J Med (2001) 345(19):1378–87. doi: 10.1056/NEJMoa010874 11794170

[47] Bartelink H Maingon P Poortmans P Weltens C Fourquet A Jager J. Whole-breast irradiation with or without a boost for patients treated with breast-conserving surgery for early breast cancer: 20-year follow-up of a randomised phase 3 trial. Lancet Oncol (2015) 16(1):47–56. doi: 10.1016/S1470-2045(14)71156-8 25500422

[48] Dong J Yang Y Han D Zhao Q Liu C Sun H. Hypofractionated simultaneous integrated boost radiotherapy versus conventional fractionation radiotherapy of early breast cancer after breast-conserving surgery: clinical observation and analysis. Technol Cancer Res Treat (2021) 20:153303382110647. doi: 10.1177/15330338211064719 PMC8671664 34898315

[49] Rockson SG. Precipitating factors in lymphedema: myths and realities. Cancer (1998) 83(12 Suppl American):2814–6. doi: 10.1002/(SICI)1097-0142(19981215)83:12B+<2814::AID-CNCR31>3.0.CO;2-E 9874403

[50] McLaughlin SA Staley AC Vicini F Thiruchelvam P Hutchison NA Mendez J. Considerations for clinicians in the diagnosis, prevention, and treatment of breast cancer-related lymphedema: recommendations from a multidisciplinary expert ASBrS panel. Ann Surg Oncol (2017) 24(10):2818–26. doi: 10.1245/s10434-017-5982-4 28766232

[51] Sayegh HE Asdourian MS Swaroop MN Brunelle CL Skolny MN Salama L. Diagnostic methods, risk factors, prevention, and management of breast cancer-related lymphedema: past, present, and future directions. Curr Breast Cancer Rep (2017) 9(2):111–21. doi: 10.1007/s12609-017-0237-8 PMC5590641 28894513

[52] Ridner SH Montgomery LD Hepworth JT Stewart BR Armer JM. Comparison of upper limb volume measurement techniques and arm symptoms between healthy volunteers and individuals with known lymphedema. Lymphology (2007) 40(1):35–46. 17539463

[53] Rastogi K Jain S Bhatnagar AR Bhaskar S Gupta S Sharma N. A comparative study of hypofractionated and conventional radiotherapy in postmastectomy breast cancer patients. Asia Pac J Oncol Nurs (2018) 5(1):107–13. doi: 10.4103/apjon.apjon_46_17 PMC5763427 29379842

[54] Chitapanarux I Klunklin P Pinitpatcharalert A Sripan P Tharavichitkul E Nobnop W. Conventional versus hypofractionated postmastectomy radiotherapy: a report on long-term outcomes and late toxicity. Radiat Oncol (2019) 14(1):175. doi: 10.1186/s13014-019-1378-x 31610801 PMC6790998

[55] Offersen BV Alsner J Nielsen HM Jakobsen EH Nielsen MH Krause M. Hypofractionated versus standard fractionated radiotherapy in patients with early breast cancer or ductal carcinoma *In situ* in a randomized phase III trial: the DBCG HYPO trial. J Clin Oncol (2020) 38(31):3615–25. doi: 10.1200/JCO.20.01363 32910709

[56] Offersen BV Alsner J Nielsen HM Jakobsen EH Nielsen MH Stenbygaard L. Partial breast irradiation versus whole breast irradiation for early breast cancer patients in a randomized phase III trial: the Danish breast cancer group partial breast irradiation trial. J Clin Oncol (2022) 40(36):4189–97. doi: 10.1200/JCO.22.00451 35930754

[57] Jagsi R Griffith KA Boike TP Walker E Nurushev T Grills IS. Differences in the acute toxic effects of breast radiotherapy by fractionation schedule: comparative analysis of physician-assessed and patient-reported outcomes in a Large multicenter cohort. JAMA Oncol (2015) 1(7):918–30. doi: 10.1001/jamaoncol.2015.2590 26247417

[58] Kunkler I. The evolving role of whole breast hypofractionation in older patients with early breast cancer. Semin Radiat Oncol (2022) 32(2):155–8. doi: 10.1016/j.semradonc.2021.11.006 35307117

[59] Pinzi V Fariselli L Jereczek-Fossa BA. Altered fractionation in radiation therapy for breast cancer in the elderly: are we moving forward? Transl Cancer Res (2020) 9(Suppl 1):S217–27. doi: 10.21037/tcr.2019.09.39 PMC8797773 35117965

