# Supplementary material for: A phase II randomized clinical trial to assess toxicity and quality of life of breast cancer patients with hypofractionated versus conventional fractionation radiotherapy with regional nodal irradiation in the context of COVID-19 crisis
Source: Front Oncol. 2023 Jun 14;13:1202544. doi: 10.3389/fonc.2023.1202544 (PMC10301738; doi:10.3389/fonc.2023.1202544)

Supplementary Material

A Phase II Randomized Clinical Trial to Assess Toxicity and Quality of Life of Breast Cancer Patients with Hypofractionated Versus Conventional Fractionation Radiotherapy with Regional Nodal Irradiation

Gabriel Oliveira Bernardes Gil1*, Warne Pedro de Andrade [2], Paulo Henrique Costa Diniz[3], Farley Soares Cantidio[4], Izabella Nobre Queiroz[5], Maria Luísa Braga Vieira Gil[6], Conceição Aparecida Medeiros Almeida[7], Paola Palmer Reis Caldeira[8], Marcos Regalin[9], Agnaldo Lopes Silva-Filho[10]*

Corresponding Author: gabrielbgil@gmail.com

1 Supplementary Data

Baseline characteristics

Group	CF-RT (n=33)	HF-RT (n=53)
Age (years)		
Mean	56	58
Ethnicity		
White	10 (30%)	22 (41%)
Mixed ethnic group	12 (37%)	23 (44%)
Black	11 (33%)	8 (15%)
Education degree		
Illiterate	2 (6%)	4 (8%)
Elementary school	17 (52%)	25 (47%)
High school	11 (33%)	18 (34%)
College education	3 (9%)	6 (11%)
Breast laterally		
Right	19 (58%)	21 (40%)
Left	14 (42%)	32 (60%)
AJCC global staging*		
II	21 (64%)	39 (74%)
III	12 (33%)	14 (26%)
Histology		
IDC	28 (85%)	43 (81%)
Others	5 (15%)	10 (19%)
Tumor size (cm)		

T1	12 (36%)	17 (32%)
T2	13 (40%)	26 (49%)
T3	8 (24%)	10 (19%)
Breast Volume (cc)		
Mean	1196 (SD 368)	1224 (SD 260)
Molecular subtype		
HER2/neu+, ER- PR-	1 (3%)	3 (6%)
Luminal A	9 (28%)	15 (28%)
Luminal B	14 (42%)	20 (38%)
HER2/neu+, HR+	5 (15%)	9 (17%)
Basal-like	4 (12%)	6 (11%)
Surgery and reconstruction		
Conservative	24 (73%)	38 (72%)
Mastectomy	9 (27%)	15 (28%)
Breast reconstruction		
Breast implant	3 (9%)	6 (11%)
Autologous tissue	1 (3%)	3 (6%)
No	5 (15%)	6 (11%)
Axillary surgery		
Axillary dissection	15 (45%)	33 (62%)
Sentinel lymph node	18 (55%)	20 (38%)
Number of axillary nodes resected (axillary dissection)		
Mean	11	9
Number of axillary nodes resected (sentinel)		
Mean	2	2
Neoadjuvant/adjuvant chemotherapy		
Yes	25 (76%)	40 (75%)
Hormone therapy		
Yes	27 (81%)	42 (79%)
Internal mammary chain irradiation		
Yes	5 (15%)	7 (13%)

Abbreviations: IDC: invasive ductal carcinoma; HER2/neu+ : Human Epidermal growth factor Receptor 2 positive; ER-: estrogen receptor negative, PR-: progesterone receptor negative; HR+: hormone receptor positive. HF-RT: hypofractionated radiation therapy; CF-RT: conventional fractionated radiation therapy; SD: standard deviation

*For patients who did not receive neoadjuvant chemotherapy, we used pathological stage because it is more accurate than clinical stage. For patients who received neoadjuvant chemotherapy, we used whichever stage was higher (clinical or pathological) to reflect the actual tumor burden.

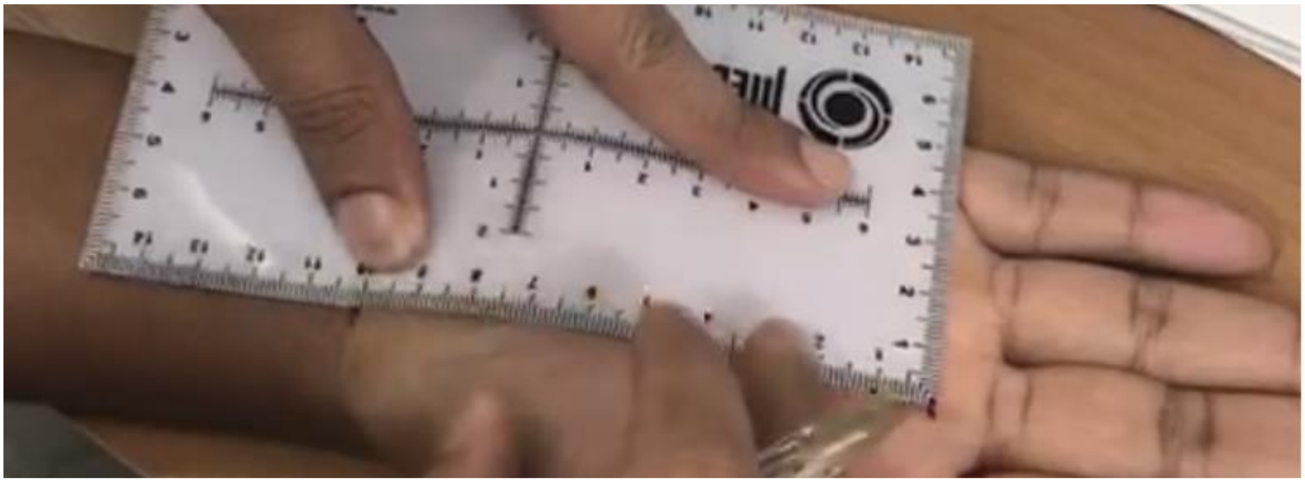

Linear measurement of the height of truncated cones. Evaluation of lymphedema with a rigid millimeter ruler (Author's photo).

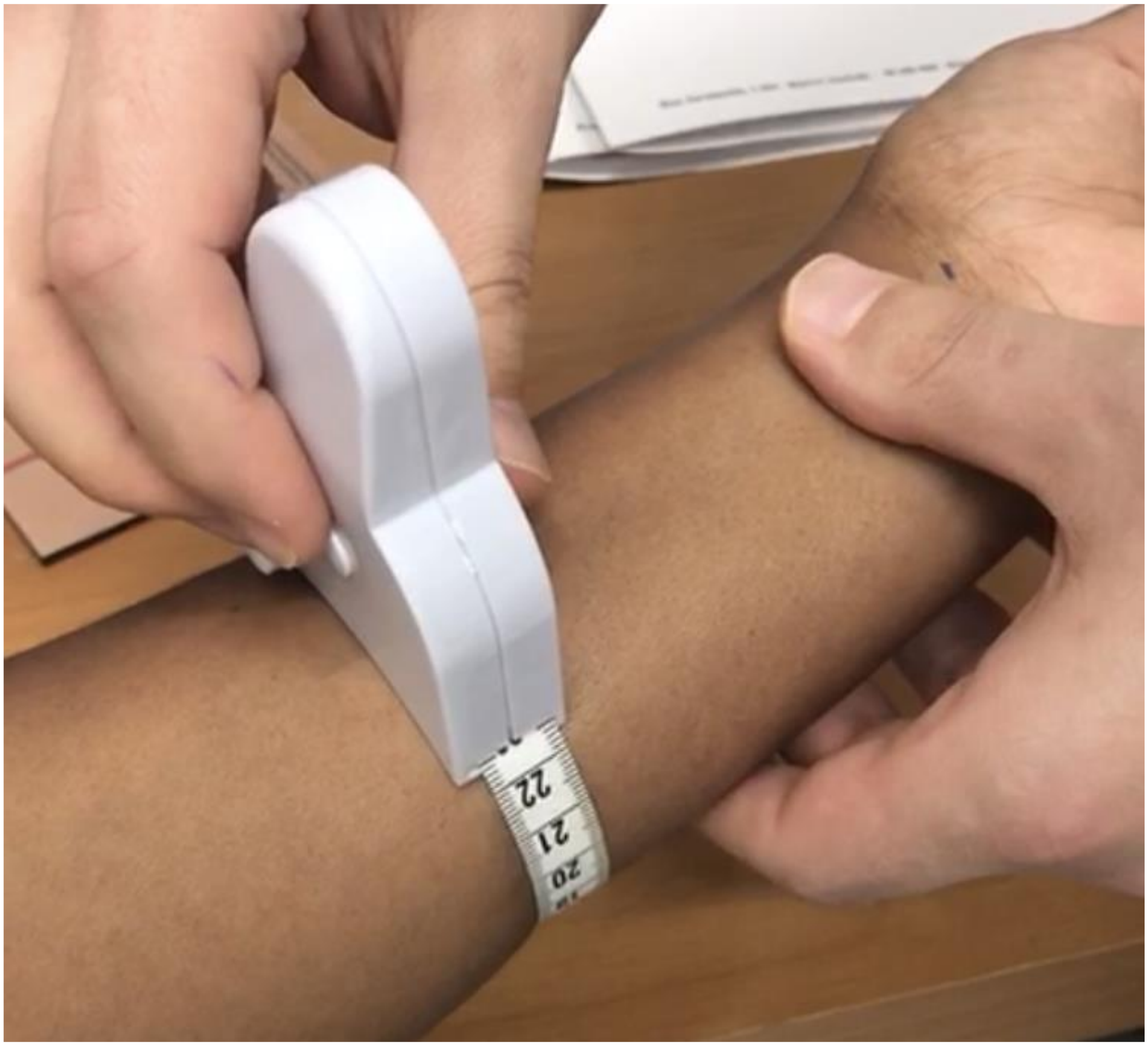

Circumferential measurement of the truncated cones with a flexible circumference measuring ruler (Author's photo).

Cosmesis Review Photos

Experimental group (HF-RT)

Patient 01 (breast-conservative surgery) – From left to right: at baseline, at the end of radiotherapy, and 6 months after treatment (Author's photo).

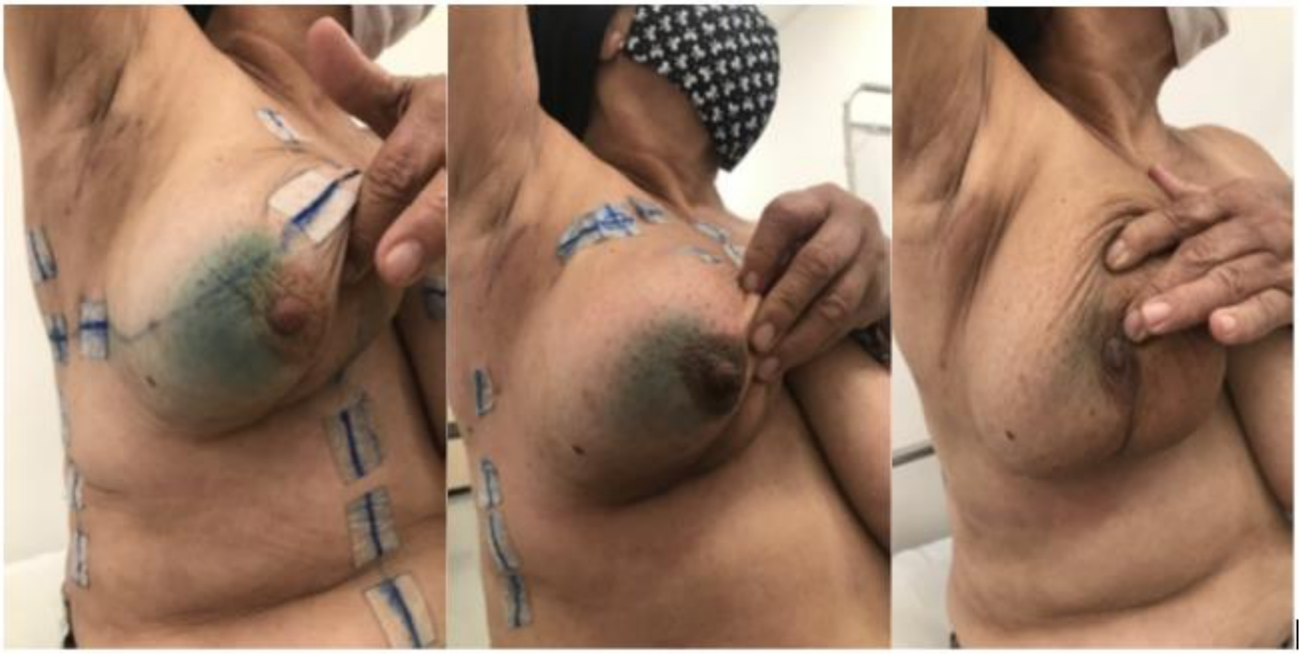

Patient 02 (breast prothesis) – From left to right: at baseline, at the end of radiotherapy, and 6 months after treatment (Author's photo).

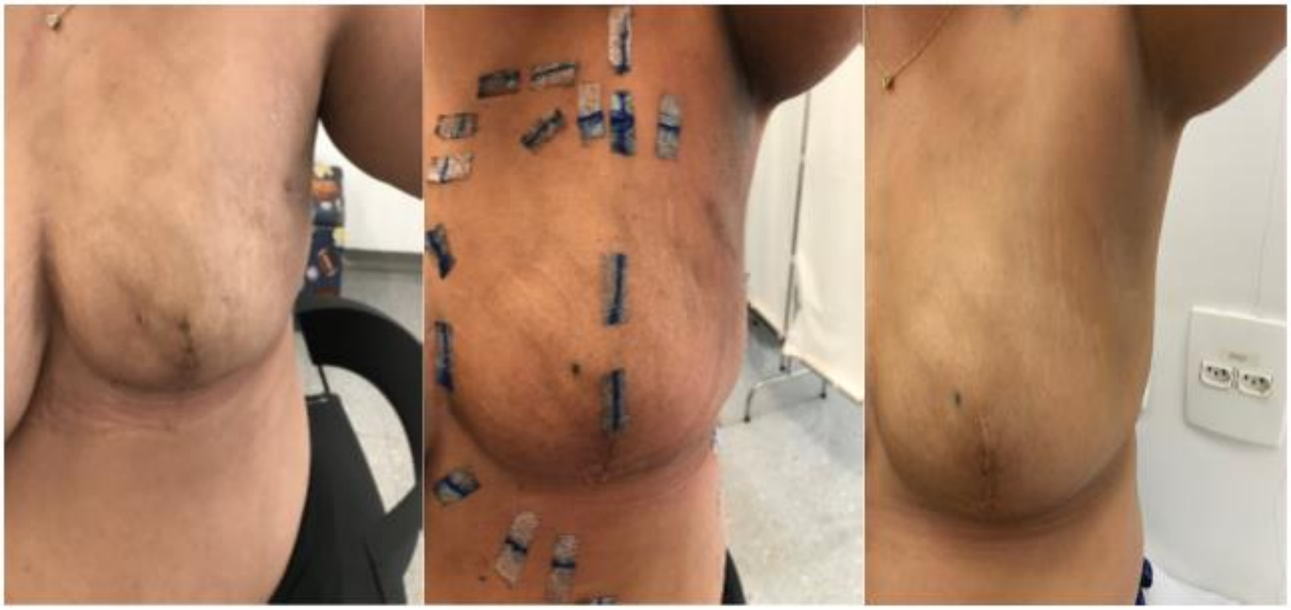

Patient 03 (mastectomy) – From left to right: at baseline, at the end of radiotherapy, and 6 months after treatment (Author's photo).

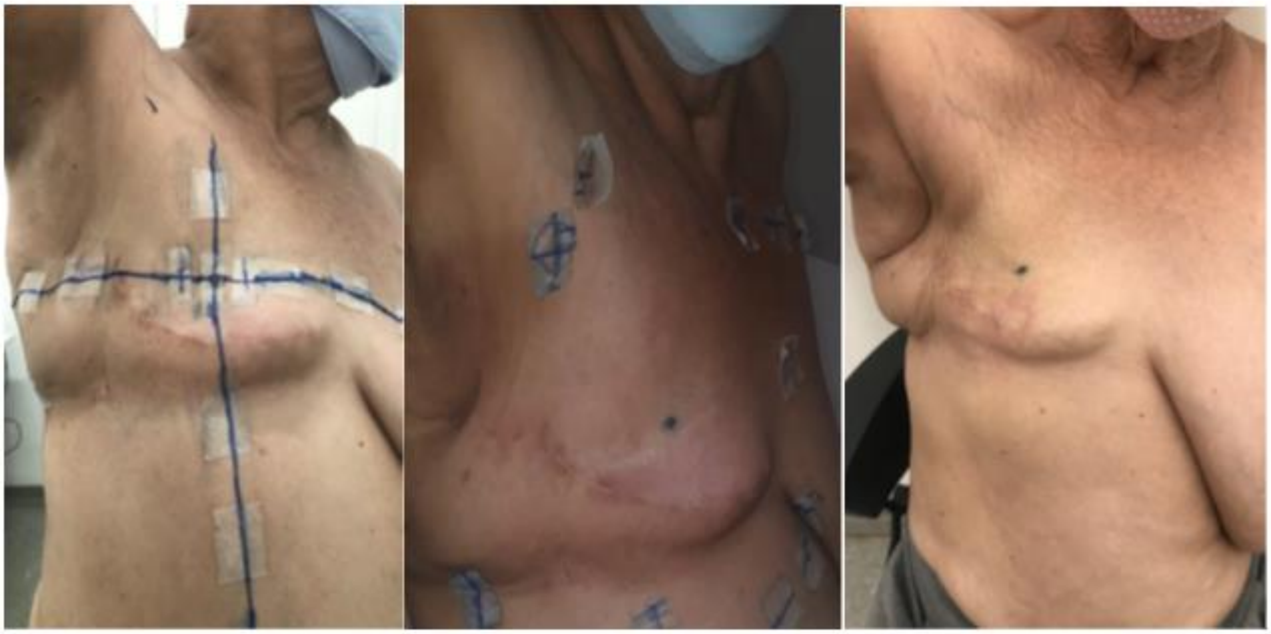

Patient 04 (breast-conservative surgery) – From left to right: at baseline, at the end of radiotherapy, and 6 months after treatment (Author's photo).

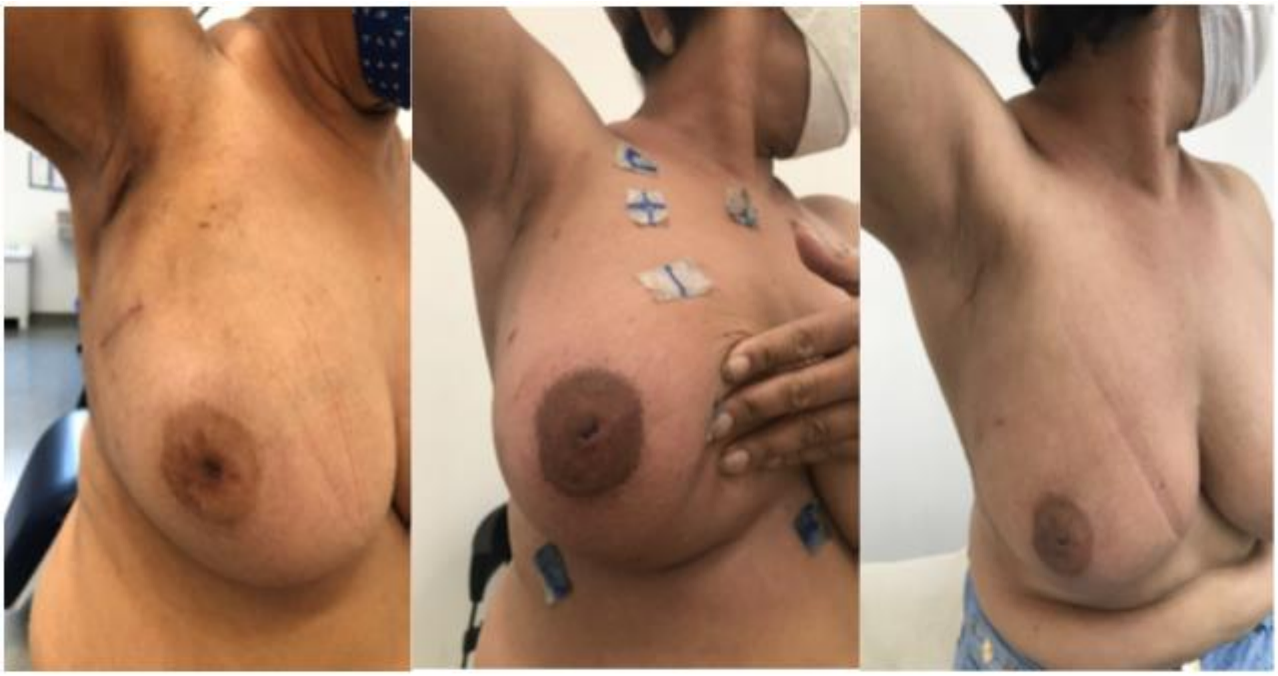

Patient 05 (expander) – From left to right: at baseline, at the end of radiotherapy, and 6 months after treatment (Author's photo).

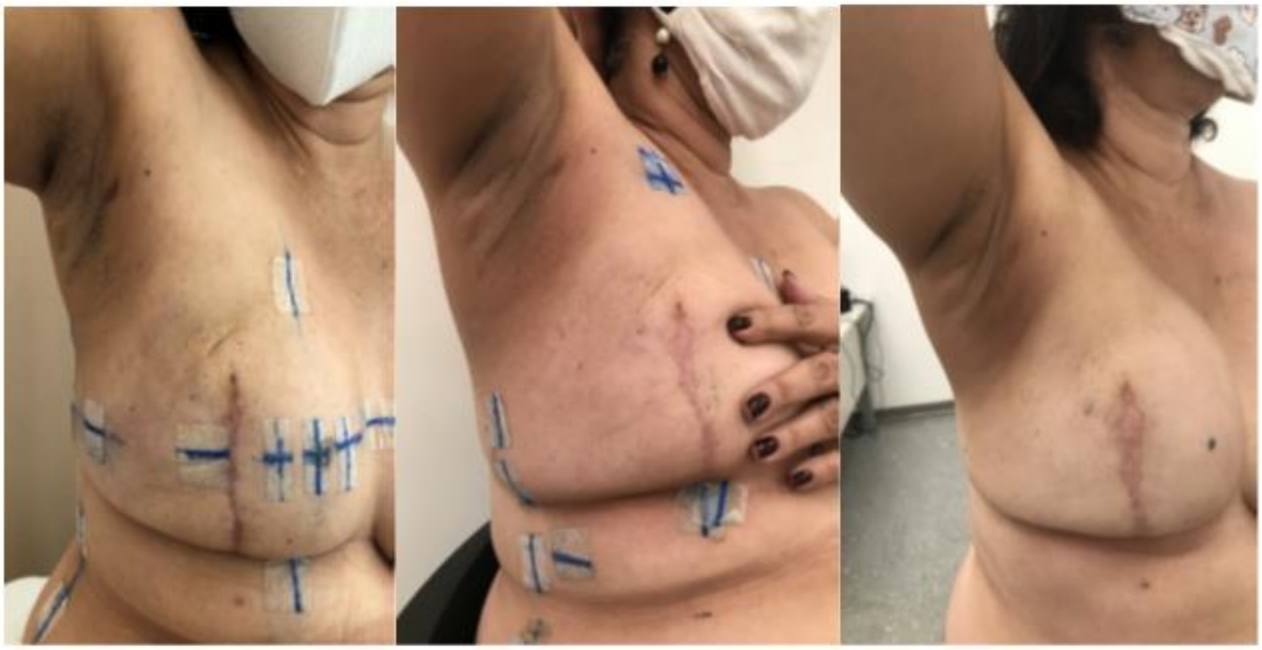

Patient 06 (mastectomy) – From left to right: at baseline, at the end of radiotherapy, and 6 months after treatment (Author's photo).

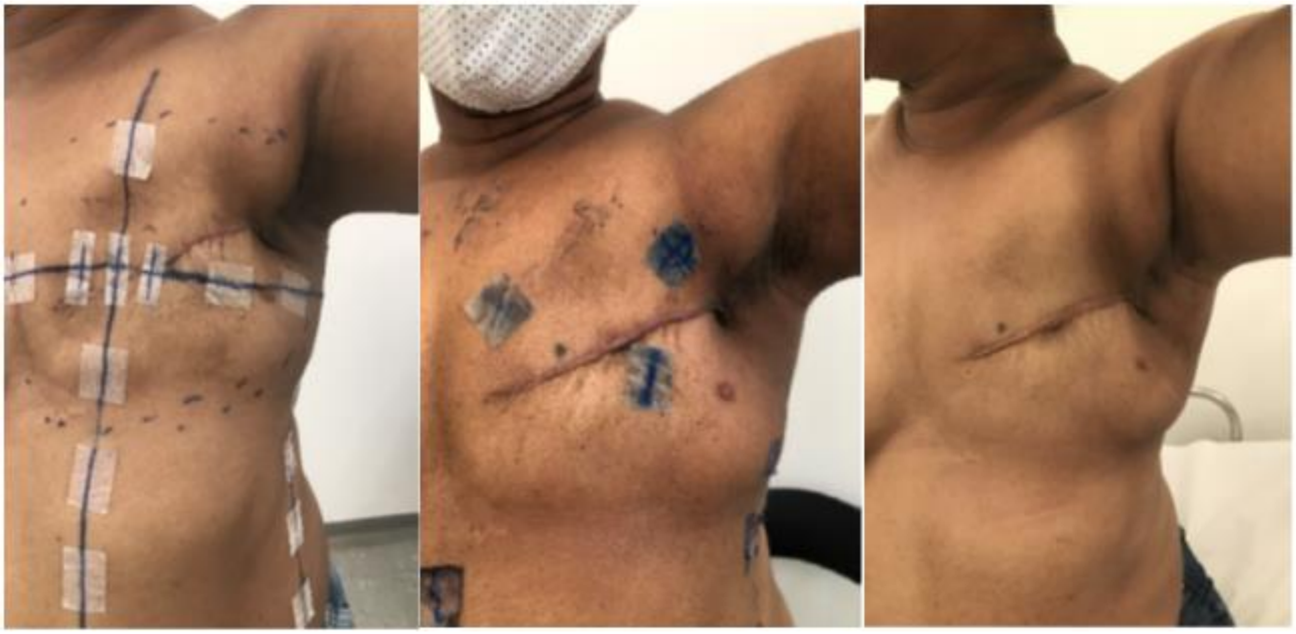

Control group (CF-RT)

Patient 07 (breast-conservative surgery) – From left to right: at baseline, at the end of radiotherapy, and 6 months after treatment (Author's photo).

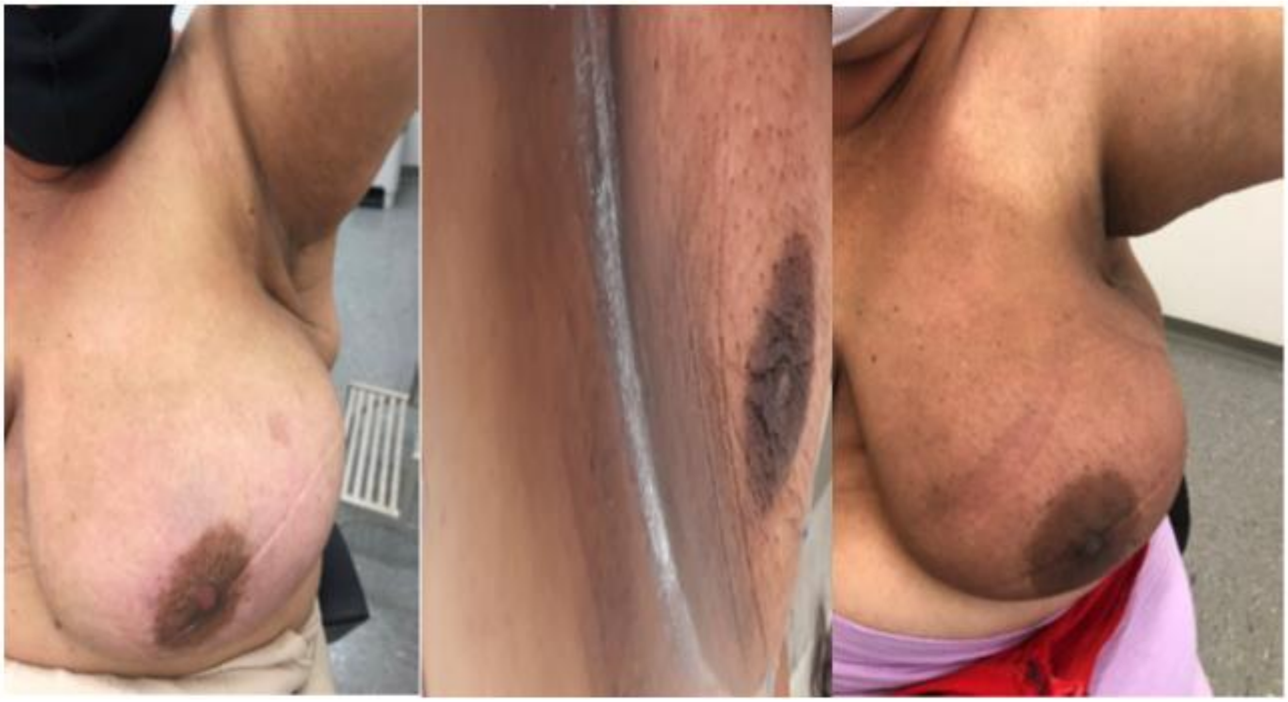

Patient 08 (breast prothesis) – From left to right: at baseline, at the end of radiotherapy, and 6 months after treatment (Author's photo).

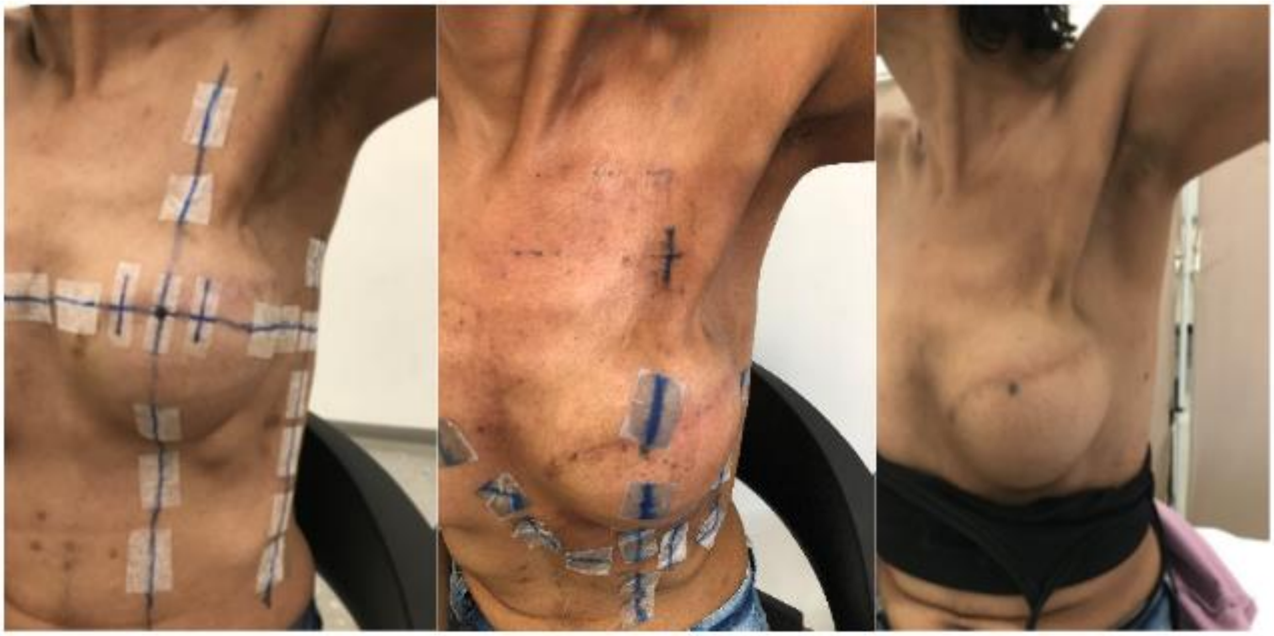

Patient 09 (mastectomy) – From left to right: at baseline, at the end of radiotherapy, and 6 months after treatment (Author's photo).

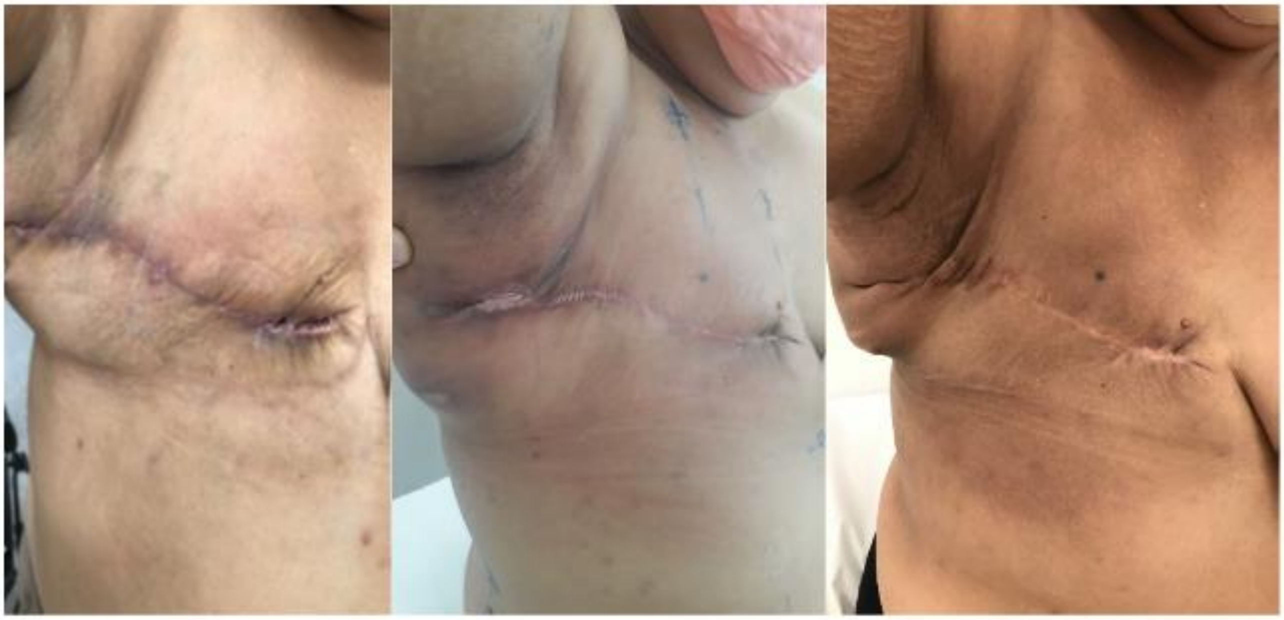

Patient 10 (breast-conservative surgery) – From left to right: at baseline, at the end of radiotherapy, and 6 months after treatment (Author's photo).

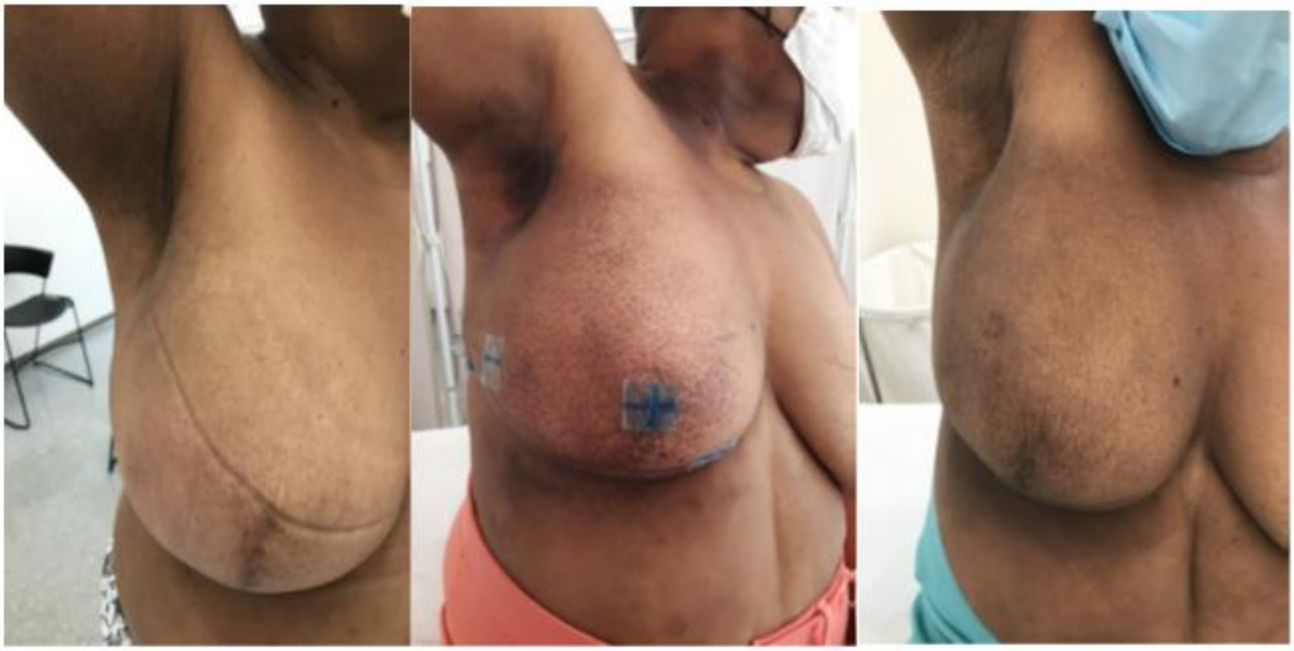

Patient 11 (expander) – From left to right: at baseline, at the end of radiotherapy, and 6 months after treatment (Author's photo).

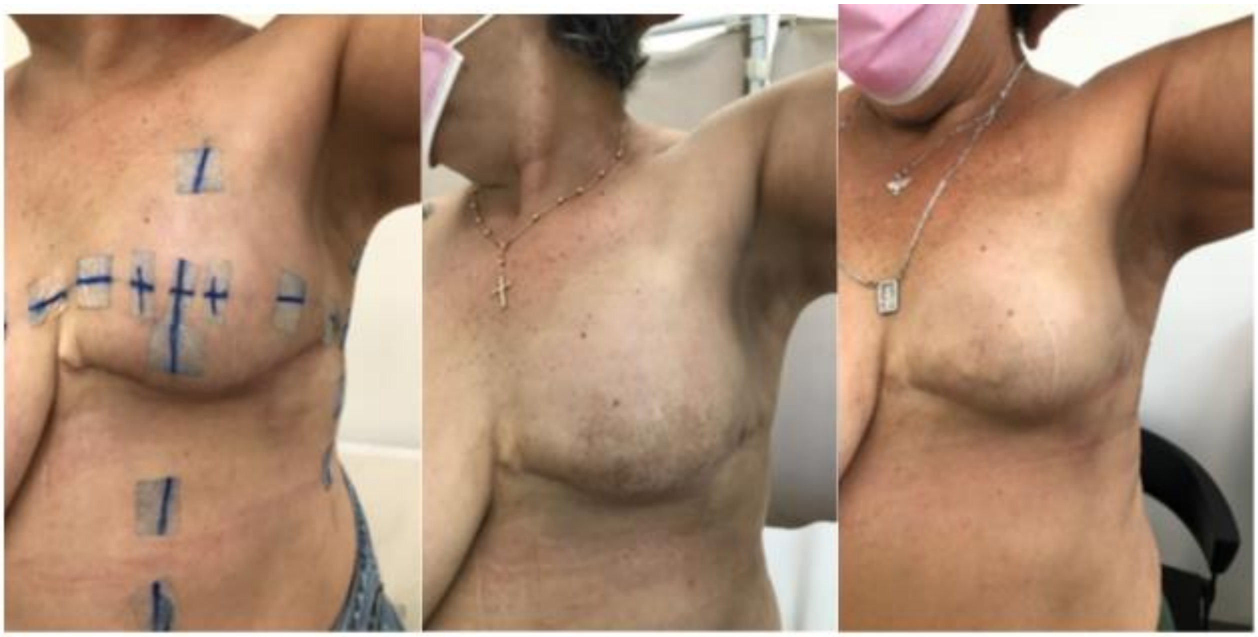

Patient 12 (mastectomy) – From left to right: at baseline, at the end of radiotherapy, and 6 months after treatment (Author's photo).

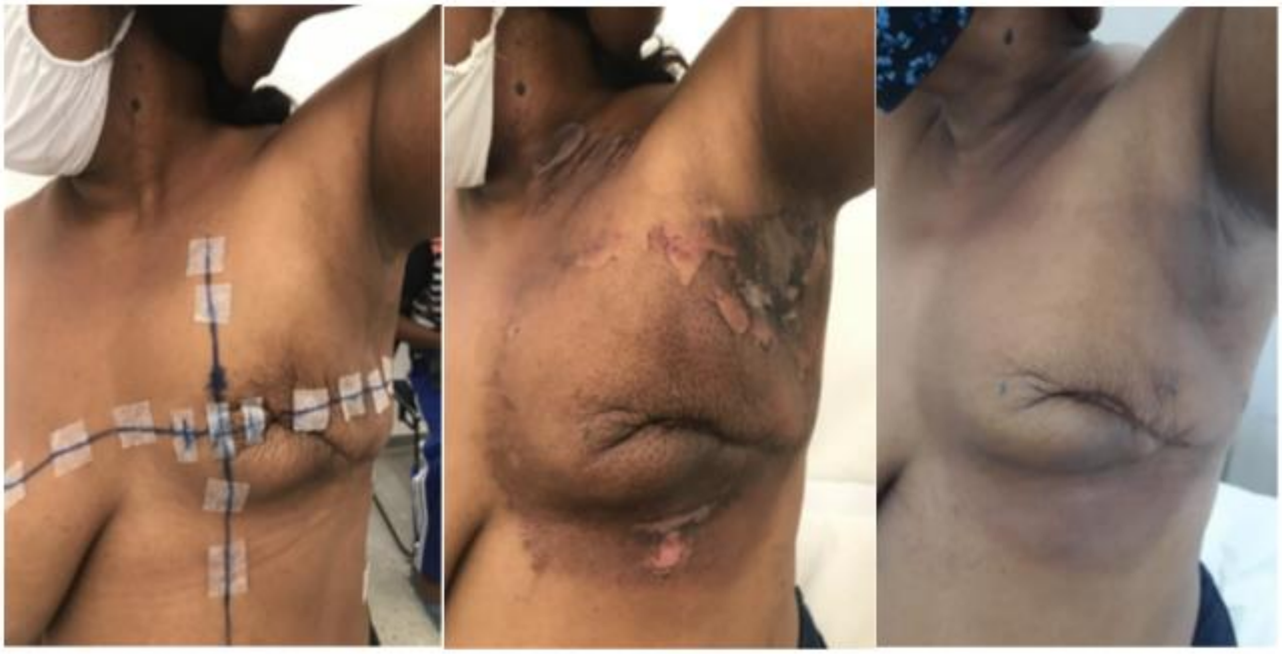

Supplement: Supplementary file 1 [file DataSheet_1.pdf]
